# First Report of Imidacloprid Biodegradation by *Cladosporium cladosporioides*: Kinetic Modeling, Transformation Products, and Toxicity Reduction

**DOI:** 10.3390/jof12100724

**Published:** 2026-09-27

**Authors:** Melissa Celeste Aguirralde, Cintia Anabela Mazzucotelli, Ignacio Durruty, Débora Nercessian, Débora Jesabel Pérez, Carla Salvio, Marcelo Clemente Sosa Morales, Erika Alejandra Wolski

**Affiliations:** 1 Instituto de Ciencia y Tecnología en Alimentos y Ambiente (INCITAA), Facultad de Ingeniería, Universidad Nacional de Mar del Plata, J.B. Justo 4302, Buenos Aires 7600, Argentina; 2 Comisión de Investigaciones Científicas (CIC), Ministerio de Ciencia, Tecnología e Innovación de Buenos Aires, La Plata 1900, Argentina; 3 Consejo Nacional de Investigaciones Científicas y Técnicas (CONICET), CCT-Mar del Plata, Buenos Aires 7600, Argentina; 4 Instituto de Investigaciones Biológicas (IIB), CONICET, Universidad Nacional de Mar del Plata, Funes 3250 4to Nivel, Buenos Aires 7600, Argentina; 5 Facultad de Ciencias Agrarias, Universidad Nacional de Mar del Plata, Ruta Nacional N° 226, Km 73.5, Balcarce 7620, Argentina; 6 Instituto de Investigaciones en Ciencia y Tecnología de Materiales (INTEMA)-CONICET, Facultad de Ingeniería, Universidad Nacional de Mar del Plata, Buenos Aires 7600, Argentina

**Keywords:** neonicotinoids, insecticides, fungi, biodegradation, metabolites, toxicity

## Abstract

This study reports, for the first time, the aerobic degradation of imidacloprid by a native fungal strain of *Cladosporium cladosporioides*, isolated and identified using ITS region sequencing (ITS1/ITS4). Degradation experiments were conducted in aqueous batch cultures supplemented with glucose using initial imidacloprid concentrations of 20, 40, and 100 mg L^−1^. After 10 days, degradation efficiencies reached 71%, 56%, and 30%, respectively, with a maximum degradation rate of 2.66 mg L^−1^ d^−1^ at 40 mg L^−1^. Growth inhibition by imidacloprid was accurately described by the inhibited Monod model. Laccase activity was strongly correlated with imidacloprid degradation kinetics. Metabolites including 5-hydroxyimidacloprid, imidacloprid olefin, and 6-chloronicotinic acid were identified using LC-MS/MS and FTIR analyses, suggesting a plausible metabolic degradation pathway. Ecotoxicological evaluation using *Eisenia fetida* avoidance tests indicated reduced toxicity of transformation products compared to the parent compound. These findings lay the groundwork for the application of *C. cladosporioides*, suggesting it may have potential for the fungal-based treatment of pesticide-contaminated aqueous environments and agricultural effluents.

## 1. Introduction

Neonicotinoid insecticides (NEOs) are among the most extensively used insecticides worldwide because of their high efficacy at low doses (0.3 mg L^−1^) and systemic activity [1]. However, their widespread application has resulted in extensive contamination of agricultural soils and aquatic environments, raising increasing ecological and human health concerns.

Neonicotinoids were introduced as safer alternatives to organophosphate and carbamate insecticides [2,3,4]. However, increasing evidence has demonstrated adverse effects on non-target organisms, including pollinators, aquatic organisms, wildlife, and humans [4,5,6,7,8]. These impacts, together with their widespread occurrence in the environment, have raised growing concern about NEO contamination and highlighted the need for effective treatment of agricultural wastewaters before environmental discharge.

Imidacloprid, the most widely used neonicotinoid insecticide worldwide, is highly soluble in water and exhibits relatively low adsorption to soils, properties that facilitate its transport and dispersion in the environment [7]. Consequently, it has been frequently detected in surface waters, groundwater, and agricultural soils worldwide [9,10], including soils without recent pesticide applications in Buenos Aires Province, Argentina, where concentrations of 14.25 µg kg^−1^ have been reported [11]. Although generally considered a low-persistence insecticide, increasing evidence indicates that imidacloprid can persist in soils for years and accumulate following repeated applications [9,12]. For example, Goulson [13] reported that repeated seed treatments increased soil concentrations from 6–8 to 18–60 ng g^−1^ over a six-year period.

Various methods of remediation exist for NEOs, including physical (adsorption), chemical (oxidation, hydrolysis, and illumination), and biological (microbial degradation) processes [14]. Adsorption merely transfers the problem to another matrix that must then be treated. Chemical methods are rapid. However, it should be noted that certain methods may not achieve complete removal of the toxic material, and some catalysts can be costly. Although imidacloprid can undergo photodegradation, this process is dependent on light penetration into the soil, pH levels, temperature, and the amount of organic matter present [8]. In contrast, biodegradation is cost-effective, environmentally friendly, and ecologically beneficial [4,14]. The main challenges associated with biodegradation are the formation of intermediates that are more lethal than the original molecule and scaling-up issues [14]. These issues could be resolved with further toxicity and scaling-up studies.

To date, numerous bacterial strains capable of degrading imidacloprid have been isolated, including species of *Tepidibacillus*, *Bacillus*, *Sphingobacterium*, and *Agrobacterium* [1,4,8,15,16]. In contrast, reports on fungal degradation remain scarce [17,18,19,20,21]. Owing to their extracellular oxidative enzymes and broad substrate specificity, fungi are promising candidates for the biodegradation of a wide range of organic pollutants. However, only a limited number of fungal taxa have been explored for imidacloprid degradation, despite the vast diversity of fungal species with potential bioremediation applications [22].

Previous studies have demonstrated imidacloprid degradation by filamentous fungi such as *Aspergillus terreus* YESM3; a consortium comprising *Aspergillus oryzae* and *Trichoderma longibrachiatum*; *Trametes versicolor*; *Phanerodontia chrysosporium*; and *Phanerochaete chrysosporium* [17,18,19,20,21]. Nevertheless, most of these studies were performed using collection strains, whereas only two investigated native isolates obtained from contaminated soils or agricultural wastewater [17,18]. Native fungi may represent more suitable candidates for field bioremediation because they are already adapted to contaminated environments.

Another important knowledge gap concerns the degradation kinetics of imidacloprid by fungi. To our knowledge, only one study has reported kinetic parameters for fungal degradation [19]. Such information is essential for developing predictive models that integrate contaminant removal and fungal growth, providing a rational basis for process design and scale-up of fungal bioremediation technologies.

Although fungal biodegradation often reduces the toxicity of neonicotinoids, some transformation products may be more toxic than the parent compound [22]. Therefore, evaluating the toxicity of degradation products is essential to assess the environmental safety of bioremediation processes. However, only a few studies have investigated the toxicity of fungal transformation products. For example, metabolites generated by *Phanerochaete chrysosporium* showed organism-dependent toxicity, whereas those produced by *Trametes versicolor* slightly increased toxicity in the Microtox assay [19,20]. In addition, earthworm avoidance behavior has been shown to be a more sensitive indicator of imidacloprid-contaminated soils than conventional acute toxicity tests, supporting the use of *Eisenia fetida* as a relevant ecotoxicological model [23].

Therefore, the objectives of this study were to isolate native soil fungi capable of degrading imidacloprid, identify the most efficient strain, characterize its degradation kinetics, investigate the metabolic transformation pathway, and evaluate the toxicity of the resulting metabolites using *Eisenia fetida*. This information contributes to the development of fungal-based bioremediation technologies for pesticide-contaminated environments.

## 2. Materials and Methods

### 2.1. Chemicals and Reagents

A high-purity (≥98%) analytical standard of imidacloprid was used as an internal standard (IS) for quantitative analysis, and laccase mediator 2,2-azino-bis (3-ethylbenzothiazoline-6-sulfonic acid) diammonium salt (ABTS, 98%) was used for quantitative activity determination. Both were purchased from Sigma-Aldrich (St. Louis, MO, USA). Acetonitrile (>98%), methanol, formic acid, and ammonium acetate were HPLC grade.

For degradation assays, commercial imidacloprid 35% (Spingard-Vertisoles, Entre Ríos, Argentina) was used. In order to avoid interference from excipients and other additives of the commercial product on the HPLC column, extraction and purification of the active ingredient was performed. For this purpose, 200 mL of commercial imidacloprid (35%) was mixed with 200 mL of dichloromethane (>99.8%), shaken vigorously, and left to stand until the two phases separated. The organic phase was then collected and concentrated using a rotary evaporator (25 °C) until dry. It was subsequently dissolved in 200 mL of acetonitrile (HPLC-grade). The solution obtained was then quantified using LC-DAD (see Section 2.7). Once its concentration was determined, it was used for subsequent degradation assays.

### 2.2. Microorganism Isolation and Growth Conditions

Soil samples were collected from the intensive strawberry commercial crops (37°53′46.1″ S 57°45′34.4″ W), where imidacloprid had been applied at least once per crop cycle, i.e., 2 times per year since 2017 (personal communication with the manager of the sampled field). Four composite soil samples were collected from two sampling points within the same field. Samples were taken from 0 to 5 cm depth in the soil profile and placed in sterile plastic bags. They were stored refrigerated at 5 °C until immediate processing in the laboratory. Twenty grams of fresh soil was taken and suspended in 180 mL of sterile peptone water for serial dilutions. The dilutions were seeded on potato dextrose agar (PDA) medium supplemented with 10 mg L^−1^ of imidacloprid to select potential degrading strains. The plates were incubated for 7 days in the dark at 25 °C. The resulting fungal colonies were isolated, purified, and transferred to PDA media containing increasing concentrations of imidacloprid (50, 150, 500, and 1000 mg L^−1^).

### 2.3. Imidacloprid Extraction from Soil Samples

The extraction process was executed in accordance with Moghaddam et al. [24], with minor adjustments. Briefly, 10 g of soil (dry weight) was placed in a 50 mL polypropylene centrifuge tubes, and 30 mL of extraction solution (acetonitrile: methanol: water at a volume ratio of 3:3:2) was added. The samples were shaking at 150 rpm for 2 h and centrifuged at 8000 rpm for 15 min. The extraction was repeated twice, and the organic phases collected were combined and concentrated using a rotary evaporator (30 °C) until dry. Subsequently, the resulting residue was dissolved in 1 mL of HPLC-grade acetonitrile, then filtered through a PTFE (polytetrafluoroethylene) membrane (0.22 µm pore size), and analyzed using LC-DAD.

### 2.4. Strain Identification

DNA extraction for each of the isolates was performed following the protocol of Huanca-Mamani et al. [25]. The DNA was quantified using a NanodropTM 2000 (Thermo Scientific, Waltham, MA, USA) and stored at −20 °C.

For the amplification of the ITS1-5.8 S-ITS2 region, primers ITS1 (5‘-TCCGTAGGTGAACCTGCGG-3′) and ITS4 (5‘-TCCTCCGCTTATTGATATATGC-3′) were used. Reactions were performed in a total volume of 25 µL containing 2.5 µL of 10X Reaction Buffer, 1.5 µL of 25 mM MgCl_2_, 1.5 µL mix dNTPs (10 mM), 1 μL of each primer (10 μM), 0.3 μL of Taq DNA Polymerase High Fidelity (5 U µL^−1^), and 2 μL of DNA (100 ng µL ^−1^) from each isolate. Nuclease-free water replaced the template DNA in the negative controls. PCR reaction was as follows: initial DNA denaturation step of 4 min at 94 °C; followed by 35 cycles of denaturation at 94 °C for 30 s, annealing at 54 °C for 45 s, and extension at 72 °C for 1 min; and a final extension at 72 °C for 5 min.

Polymerase chain reaction products were purified using the PuriPrep-GP Kit (Tandil, Buenos Aires, Argentina). DNA quality was verified by determining the ratio of A260/A280 and A260/A230 on a NanoDrop. Sequencing was carried out at Macrogen, Inc. (Seoul, Republic of Korea). The sequences obtained were analyzed using the Blast tool of the National Center for Biotechnology Information [26]. For phylogenetic analysis, the MEGA program version 11 (Penn State University, University Park, PA, USA) was used, applying the Maximum Likelihood (ML) method with the Tamura-Nei substitution model. Branch strength was evaluated using the bootstrap method with 1000 replicates. Ten representative sequences of *Cladosporium* and one sequence of *Penicillium chrysogenum*, selected as an outgroup, were included in the analysis in order to root the tree.

### 2.5. Degradation Batch Experiments

To assess the biodegradation of imidacloprid, strains able to grow and tolerate high concentrations of imidacloprid were inoculated directly from the PDA plate (five PDA agar disks of 4 mm diameter containing the fungal mycelia) into 150 mL minimal mineral salt medium (LMS) supplemented with glucose (10 g L^−1^) as a carbon source for fungal growth and imidacloprid (20 mg L^−1^). The LMS contained 1000 mL deionized water, 0.1 g MgSO_4_·7H_2_O, 0.1 g K_2_HPO_4_, 1 g NaNO_3_, 1 g KCl, and 25 μL of trace element solution (in mg L^−1^: MnSO_4_ 15.4, FeCl_3_ 40, ZnSO_4_·7H_2_O 6.3, CuSO_4_·5H_2_O 2.5, NH_4_·MO_7_·O_2_·4H_2_O 0.5). The pH was adjusted to 6.0. Then flasks were incubated in the dark at 25 °C with shaking at 150 rpm. The same procedure was followed for the control (uninoculated). The first assays were carried out during 20 days of incubation, and subsequent assays were adjusted to an incubation period of 10 days.

Samples from the liquid media were taken and analyzed daily for the presence of imidacloprid using LC-DAD.

For kinetic assays, the LMS medium was supplemented with glucose (10 g L^−1^) as a carbon source and 20, 40, and 100 mg L^−1^ of imidacloprid. The exact initial concentrations of imidacloprid measured using LC-DAD were 17.25 ± 0.04, 42.86 ± 4.53, and 99.19 ± 13.46 g L^−1^, respectively.

All experiments were carried out in triplicate. In each experiment, three replicates were carried out, and the results show the mean value of three independent experiments.

To verify that the decrease in imidacloprid was due to biodegradation and not to physical adsorption to the fungal mycelium, a control was carried out, killing the fungus by heat. For this, LMS (150 mL) was inoculated with 5 PDA disks of 4 mm diameter containing the fungal mycelia, and then it was autoclaved at 121 °C for 30 min. Once the autoclaving process was completed, imidacloprid was added to the medium (at a 20 mg L^−1^ final concentration). The culture was incubated for 10 days at 25 °C with shaking (150 rpm). Samples were taken every 48 h and filtered using a 0.22 μm PTFE (polytetrafluoroethylene) membrane for subsequent LC-DAD analysis.

Initial degradation rates were calculated as Δ*C*/Δ*t*, where *C* is imidacloprid concentration in mg L^−1^ and *t* is time in days.

### 2.6. Fungal Growth and Biomass Determination

Fungal growth was determined by measuring mycelium dry weight. The test was carried out by sacrifice. Every two days, triplicate Erlenmeyer flasks were sacrificed, and the fungal mycelium was filtered onto a Whatman GF/A filter (grade 1), rinsed twice with distilled water, and dried at 100 °C until constant weight. Biomass was calculated as mg of dry weight per volume of reactor (L).

### 2.7. Imidacloprid Quantification

The concentration of imidacloprid was measured using chromatography. The LC-DAD equipment consisted of a Shimadzu LC-20AT (Kyoto, Japan) with a diode array detector (SPD-M20A, Shimadzu, Kyoto, Japan). The separation was achieved with an analytical column Shim-pack GWS C18 (5 µm, 4.6 × 250 mm) (Shimadzu, Kyoto, Japan) by isocratic elution with a mobile phase constituted of a mixture of deionized water:acetonitrile (60:40 *v*/*v*). The injection volume was of 20 μL. The flow rate was maintained at 0.7 mL min^−1^, and the column compartment was kept at the temperature of 20 °C. Imidacloprid was identified by comparing their retention time with the external standard and using co-chromatography. Under the above conditions, the retention time of imidacloprid was 8.4 min. The detection of imidacloprid was performed at 270 nm. Calibration standard curves of six points (10 to 75 mg L^−1^) for imidacloprid were made in ultrapure water with methanol. For these, a stock solution of 200 mg L^−1^ of imidacloprid was prepared in HPLC-grade methanol and stored at 4 °C in darkness.

### 2.8. FTIR Analysis

The metabolites produced after degradation of imidacloprid also were analyzed using attenuated total reflectance–Fourier transform infrared (ATR-FTIR) spectra to complement the results of the LC/MS-MS. A Nicolet 6700 FT-IR (Madison, WI, USA) was used in the mid infrared region of 400–4.000 cm^−1^. All spectra were acquired with a resolution of 4 cm^−1^ and 64 scans. The samples were previously freeze-dried to prevent interference from the water signal.

### 2.9. LC/MS-MS Analysis of Transformation Products

The metabolic products, obtained after 10-day transformation by *Cladosporium cladosporioides* B1, were identified using an UPLC I-Class/XEVO TQ-XS of Triple Quadrupole MS system (Milford, MA, USA) operated in positive and negative electrospray ionization mode.

The samples were diluted 500 fold in ultrapure water in a 1.5 mL plastic tube and then vortexed for 30 s. Then, the diluted samples (1 mL) were filtered through a 0.22 µm nylon filter and dispensed into a 1.5 mL amber vial for LC-MS/MS determination.

The target analytes were separated using a C18 column (2.1 100 mm, 1.8 µm I.D., 0.3 mL min^−1^ flow rate, 5 min run time, 10 µL injections) maintained at 45 °C. The mobile phase consisted of organic-free ultra-pure water containing 0.01% formic acid and 0.1 mM of ammonium acetate (A) and HPLC-grade methanol containing 0.01% formic acid and 0.1 mM of ammonium acetate (B). The percentage of B was changed linearly as follows: 5% at 0.5 min; 100% at 3 min; 100% at 4.5 min; and 5% at 5.0 min. The transformation products were identified qualitatively using retention time (RT) and two ion products from the corresponding precursor ion. The Mass Hunter Workstation Software Version B.07.00/Build 7.0.457.0 (Agilent Technologies, Santa Clara, CA, USA) was used to process the data obtained.

Quality controls: Triplicate solvent blanks, spiked solvent control samples, and spiked tissue samples were analyzed along with each batch of homogenate samples. Blanks were monitored to identify contamination or carry over. Continuous calibration verification samples were analyzed every five samples. Calibration standard curves (0.1 to 100 µg L^−1^) for imidacloprid were made in ultrapure water with methanol.

### 2.10. Kinetic Model Development

A Monod-type kinetic model was proposed to fit and predict the biomass (*X*) growth, substrate (*S*) consumption, and imidacloprid (I) depletion simultaneously. In absence of imidacloprid, the data were fitted to the Monod kinetic model, which is depicted by the differential equations system 1 and 2, where *X* is the biomass weight (g) per volume (mL) of the culture media and *S* represents the growth substrate as glucose concentration.

(1) $$\frac{\partial X}{\partial t}=r_{X}=\mu .X=\frac{\mu_{max}.S}{Ks+S}.X$$

(2) $$\frac{\partial S}{\partial t}=r_{S}=-\frac{1}{Y_{\frac{X}{S}}}\mu .X=-\frac{1}{Y_{\frac{X}{S}}}\frac{\mu_{max}.S}{Ks+S}.X$$

In the presence of imidacloprid, a growth Monod model with inhibition kinetics was proposed. When the consumption of imidacloprid is considered as growth-associated contaminant uptake. Therefore, the kinetic model in the presence of imidacloprid is depicted by Equation systems 2, 3 and 4, where *I* is the imidacloprid concentration.

(3) $$\frac{\partial X}{\partial t}=r_{X}=\mu ’.X=\frac{\mu_{max}}{1+\frac{I}{KI}}.\frac{S}{Ks+S}.X$$

(4) $$\frac{\partial S}{\partial t}=r_{S}=-\frac{1}{Y_{\frac{X}{S}}}\mu ’.X=-\frac{1}{Y_{\frac{X}{S}}}\frac{\mu_{max}}{1+\frac{I}{KI}}.\frac{S}{Ks+S}.X$$

(5) $$\frac{\partial I}{\partial t}=r_{I}=-Y_{\frac{I}{X}}\mu ’.X=-Y_{\frac{I}{X}}\frac{\mu_{max}}{1+\frac{I}{KI}}.\frac{S}{Ks+S}.X$$

In the context of Monod’s model, the term “μ” denotes the Monod specific growth rate, while “μ’ “ is designated as the Monod inhibited specific growth rate. It can be defined that the kinetic parameters of μ and μ’ are the maximum Monod specific growth rate (*μ_max_*), the Monod constant (*K_S_*), and the inhibition constant (*K_I_*).

The *Y_X/S_* substrate yield coefficient is indicative of the extent to which the biomass increases in proportion to the glucose. Y_I/X_ is the imidacloprid consumption yield coefficient, representing the mass of imidacloprid depleted by the mass of biomass growth (g).

The kinetic parameters (μmax, *K_S_*, and *K_I_*) were obtained with non-linear fitting of the respective rate data (*r_X_*, *r_S_*, and *r_I_*). The rate data were obtained using centered finite differences of the experimental data. The yield coefficients Y_X/S_ and Y_I/X_ were obtained as the scope of linear fitting of X versus S data and I versus X data, respectively. Once the parameters were fixed, profiles were modeled using a fourth-order Runge–Kutta algorithm coupled to the regression in order to integrate the differential equations simultaneously.

### 2.11. Determination of Laccase Activity

Laccase activity was determined spectrophotometrically by measuring the oxidation of 2,2′-azino-bis(3-ethylbenzothiazoline)-6-sulfonic acid (ABTS) at 420 nm in the liquid medium of the degradation batch culture. For this purpose, the incubation mixture contained 100 µL of sample, 200 µL of 0.5 mM ABTS, and 100 mM sodium acetate buffer (pH 5.0) in a final volume of 1 mL. The mixture was incubated at 25 °C for 3 h. A control without enzyme, consisting only of ABTS and buffer, was included. A unit of laccase activity was defined as that which generates a change in absorbance of 1.0 min^−1^ mL^−1^ at 25 °C.

### 2.12. Avoidance Behavior Assay

Specimens of *Eisenia fetida* earthworms were obtained from breeding carried out in a rearing chamber at 20 ± 2 °C and 14:10 h light:dark photoperiod. For this purpose, adult individuals (with clitellum) were placed in 30 × 40 cm, 25 cm high plastic containers with moist soil and fed with organic plant remains (fruit peels, yerba mate, and coffee grounds). Adults with well-developed clitellae and individual weight between 300 and 600 mg were selected for the assay [27]. The soil was a fine, mixed, thermic Typic Argiudoll (USDA classification) containing 5.1% organic matter; 20.9% clay, 34.9% silt, and 44.2% sand; pH 7.7; conductivity of 0.6 mmhos cm^−1^; and cation exchange capacity of 29.3 meq 100 g^−1^ [27].

Rectangular plastic containers (18 cm long by 12 cm wide and 12 cm height) provided with a perforated lid were used (Scheme 1). Each container was divided in two equal sections by a removable plastic split [28]. One half of the container received 750 g of soil control (with water), and the other half received 750 g of treatment soil. The following treatments were tested: (T1) imidacloprid (20 mg L^−1^) and (T2) biodegradation products after *C. cladosporioides* treatment. To achieve the active ingredient doses per kg of dry soil, the insecticide was diluted in distilled water, considering that the weight of soil from one hectare at 5 cm and with an apparent density of 1.2 Mg m^−3^ is equivalent to 600,000 kg. Applications of the insecticide dissolved in water were made with a manual sprayer on a thin, uniform layer of soil 5 cm high on a 50 × 50 cm nylon fabric. To ensure adequate incorporation of the product into the substrate, 20 movements were made from the sides of the fabric to the center.

After the separator was removed, ten earthworms of 290–310 mg were placed in the center of the container. The design was completely randomized with five replicates per treatment following the methodology of Amorim et al. [29]. The experiment was performed in a chamber in a 14:10 h light:dark photoperiod at a temperature of 20 ± 2° C. After 48 h, the removable split was reintroduced, and the individuals of each section were counted. The results of the counting were expressed as net response (NR or avoidance response) in percentage according to ISO [30]: NR = ((C − T)/N) x100, where C is the number of observed worms in the control soil, T is the number of worms in the imidacloprid treated soil, and N is the total number of worms [29]. A positive NR indicates avoidance of the treated soil, whereas 0% or a negative value indicates a non-response or attraction to the pesticide tested, respectively. To test significant differences between mean values an ANOVA test was performed (α = 0.05). Data normality and homogeneity of variances were always confirmed, and the analyses were done with R version 4.5.1 (R Foundation for Statistical Computing, Vienna, Austria) [31].

## 3. Results

### 3.1. Isolation and Characterization of Fungal Imidacloprid Degrading Strains

Soil samples from the intensive strawberry crop, where imidacloprid had been applied, showed an imidacloprid concentration of 0.02 ± 0.01 mg kg^−1^ of soil. From these, thirteen fungal strains were isolated. These strains were able to grow and tolerate high concentrations of imidacloprid (50, 150, 500, and 1000 mg L^−1^) in PDA plates. Analysis of the ITS region sequences in the database indicated that they all cluster within genera *Apiospora*, *Alternaria*, *Cladosporium*, *Trichoderma*, *Epicoccum,* and *Fusarium*, all from the Ascomycota division, ranging from 94% to 100% coverage (Table 1).

The ability of these isolates to degrade imidacloprid was then evaluated in batch cultures with LMS medium supplemented with glucose (5 g L^−1^) as a carbon source and with 20 mg L^−1^ of imidacloprid (value measured by LC-DAD 19.53 ± 0.22) for 20 days. Only two strains were able to degrade imidacloprid after 20 days of incubation. One of them, arbitrarily named A10, degraded 17.1 ± 6.6%, while the other, named B1, degraded 53.0 ± 15.0%.

Analysis of the ITS1–5.8S rDNA–ITS2 sequence using BLAST (version 2.17.0+, National Center for Biotechnology Information, Bethesda, MD, USA) indicated that strain B1 is highly similar to reference sequences of *C. cladosporioides* (Figure 1). In the phylogenetic tree obtained, strain B1 is grouped within the main clade of *C. cladosporioides* together with multiple sequences deposited in GenBank, with a bootstrap of 100%, which robustly confirms its identity. Strain A10 was also located within the main clade, although forming a separate subclade, suggesting possible genetic and functional differences. Other closely related species, such as *C. herbarum*, *C. tenuissimum*, and *C. halotolerans*, were clearly differentiated from the *C. cladosporioides* clade, while *Penicillium chrysogenum* was used as an outgroup, allowing the tree to be properly rooted. Based on these results, the experiments were continued with strain B1, hereafter referred to as *C. cladosporioides* B1 (PZ136811).

### 3.2. Bioegradation Assays

The degradation kinetic assay was carried out with *C. cladosporioides* B1 in the presence of 20 mg L^−1^ of imidacloprid. The kinetic results showed a maximum degradation rate of 1.42 ± 0.31 mg L^−1^d^−1^, reaching a maximum degradation of 71.43 ± 3.49% at day 10 (equivalent to 4.72 ± 0.6 mg L^−1^ of imidacloprid remaining) (Table 2, Figure 2). Higher degradation rates were observed between days 2 and 8, corresponding to the exponential growth phase of *C. cladosporioides* B1. Moreover, an adsorption control was conducted, which demonstrated that a mere 5% of imidacloprid was lost due to adsorption by the mycelium at a rate of 0.73 ± 0,23 mg L^−1^d^−1^. This outcome confirms that the dissipation of imidacloprid from the culture medium is attributable to a degradation mechanism. In addition, no degradation was observed in the abiotic control (0 ± 0.08 mg L^−1^d^−1^).

Subsequently, batch cultures were conducted in LMS medium supplemented with different initial imidacloprid concentrations (20, 40, and 100 mg L^−1^). The findings demonstrated that *C. cladosporioides* B1 exhibited the capacity to degrade all initial concentrations of imidacloprid (Table 2). However, its degradation capacity exhibited an inverse relationship with the initial imidacloprid concentration, with values of 71.43 ± 3.49%, 56.37 ± 11.71%, and 30.28 ± 9.64% observed for concentrations of 20, 40, and 100 mg L^−1^, respectively. Table 2 also shows that the presence of imidacloprid significantly increased the growth rate of *C. cladosporioides* B1 at all the initial concentrations tested. Furthermore, in the presence and absence of imidacloprid, the pH of the culture increased significantly, rising from 6.45 ± 0.13 (day 0) to 7.31 ± 0.21 and 8.25 ± 0.83 (day 10) with and without imidacloprid, respectively (unpaired *t* test with Welch’s correction, *p* < 0.05). However, on the tenth day, the increase in pH in the presence of imidacloprid was found to be significantly lower than in the absence of the insecticide.

### 3.3. Kinetic Modeling

Table 2 summarizes the kinetic parameters and yield coefficients obtained in this study. The values of µmax, K, and Y_X/S_ were estimated by fitting the experimental data from cultures grown without imidacloprid to the Monod kinetic model (Equation (1)). These parameters represent the intrinsic growth characteristics of *C. cladosporioides* B1 under non-inhibitory conditions and were used as the baseline to evaluate the effect of imidacloprid on fungal growth. Figure 3 presents the experimental data and the corresponding Monod model predictions for cultures grown in the absence of imidacloprid.

The inhibition parameters (Y_I/X_ and K_I_) were estimated by fitting the experimental data obtained at different initial imidacloprid concentrations. A single parameter set was unable to adequately describe the entire dataset; therefore, the inhibition parameters were calibrated separately for each initial imidacloprid concentration. Figure 4 compares the experimental data with the predictions of the Monod kinetic model (Equation (1), dashed lines) and the inhibited Monod kinetic model (Equation (2), solid lines). The Monod model consistently overestimated fungal growth in the presence of imidacloprid, as it does not account for inhibitory effects. In contrast, the inhibited Monod model provided a substantially better fit to the experimental data across all tested concentrations, accurately describing the reduction in biomass growth caused by imidacloprid.

From a mathematical perspective, Y_I/X_ represents the amount of imidacloprid degraded per unit of biomass produced. The increase in Y_I/X_ with increasing initial imidacloprid concentration indicates that more contaminant was degraded for each gram of biomass formed. Because the model assumes growth-associated degradation, this result suggests a strong coupling between fungal growth and imidacloprid consumption. The observed increase in Y_I/X_ may reflect the greater availability of the contaminant at higher initial concentrations and/or a reduction in biomass production caused by toxic effects, rather than an increase in fungal growth itself.

The inhibition constant (K_I_) describes the effect of imidacloprid on fungal growth. Lower K_I_ values indicate a stronger inhibitory effect on the specific growth rate (µ). Accordingly, the estimated parameters suggest that growth inhibition became more pronounced as the initial imidacloprid concentration increased. At 20 mg L^−1^, fungal growth was noticeably inhibited. In contrast, at 40 mg L^−1^, the model predicted a much stronger inhibitory effect. Interestingly, the estimated K_I_ values did not follow a single concentration-independent trend, indicating that growth inhibition alone may not fully explain the observed behavior. Instead, these results suggest that toxicity and inhibition may act simultaneously. While toxicity reduces biomass formation, it may not proportionally reduce imidacloprid consumption, leading to higher apparent Y_I/X_ values at elevated contaminant concentrations.

### 3.4. Laccase Activity

Laccases are among the oxidative enzymes most frequently involved in the fungal biodegradation of organic pollutants [32], and *Cladosporium cladosporioides* has previously been reported as a laccase-producing species [33,34]. Therefore, we investigated whether extracellular laccase activity was induced during imidacloprid degradation by *C. cladosporioides* B1. As shown in Figure 5, laccase activity increased after the onset of fungal growth, reaching its highest values during the period of maximum imidacloprid degradation rate. Although the timing of the activity peak varied slightly with the initial imidacloprid concentration, the temporal profiles of laccase activity and degradation rate closely matched across all treatments. Correlation analysis between laccase activity and the imidacloprid degradation rate (Equation (5)) yielded high coefficients of determination (R^2^ = 0.87, 0.94, and 0.94 for 20, 40, and 100 mg L^−1^, respectively), indicating an association between both variables until day 8 for all concentrations. This temporal association may indicate that laccase activity is related to the imidacloprid degradation process by *C. cladosporioides* B1. However, the observed correlation does not demonstrate a direct catalytic role of laccase in imidacloprid oxidation, and the contribution of other oxidative enzymes or indirect mechanisms cannot be excluded.

### 3.5. Transformation Products

To analyze the imidacloprid transformation products after *C. cladosporioides* B1 treatment, biodegradation assays were carried out with 40 mg L^−1^ of imidacloprid. At 0 and 10 days, the samples were analyzed using FTIR and LC-MS/MS. Imidacloprid of both qualities was detected in control and treated samples, indicating incomplete degradation. Different compounds, which are known products derived from imidacloprid transformation, were identified in the treated samples (Table 3 and Supplementary Material. When biodegradation tests were performed with high-purity (standard grade) imidacloprid, only 5-hydroxy imidacloprid was detected. However, when commercial imidacloprid was used, imidacloprid olefin and 6-chloronicotinic acid were detected in addition to 5-hydroxy imidacloprid.

FTIR analysis further supported the structural transformation of imidacloprid during fungal treatment. Compared with the spectrum of the untreated standard, the degraded sample exhibited marked spectral changes (Figure 6), including a substantial reduction of the band at approximately 1560 cm^−1^, assigned to N=N stretching within the imidazolidine ring [35], and a decrease in the band at 1355 cm^−1^ associated with N–O stretching of the nitro group [15]. In contrast, the absorption around 1000 cm^−1^, attributed to C–O stretching [36], became more intense after fungal treatment. These spectral modifications are consistent with oxidative transformation of imidacloprid and support the LC-MS/MS identification of 5-hydroxy imidacloprid as one of the major transformation products.

### 3.6. Avoidance Assay

A rapid sublethal avoidance assay was performed to compare the behavioral toxicity of the fungal degradation products with that of the parent compound (Figure 7). No mortality was observed in either treatment, resulting in a 100% survival rate. However, the net response (NR) differed significantly between treatments (*p* = 0.0196). *Eisenia fetida* exhibited a negative avoidance response (NR = −44%) toward soil amended with the fungal degradation products, indicating a preference for this substrate, whereas a clear avoidance response was observed for soil containing imidacloprid (NR = +48%). These findings suggest that the metabolites generated during degradation by *C. cladosporioides* B1 have a lower behavioral toxicity than the parent insecticide, indicating a reduction in ecotoxicological impact following fungal treatment.

## 4. Discussion

In the present work, two different fungal strains of *C. cladosporioides* capable of degrading imidacloprid, have been isolated. This is the first report describing *C. cladosporioides* as a degrader of a NEOs. Both strains were grouped in different clades within the phylogenetic tree, suggesting possible genetic and functional differences, when compared with other *C. cladosporioides* reported in the literature. These functional differences could at least explain the different degradation rates measured in this work. As can be seen in Figure 1, *C. cladosporioides* B1(PZ136811) is closest to GQ241276, which has been shown to tolerate higher concentrations of PHAs and has been reported to be able to remove high levels (800 mg L^−1^) of phenanthrene [37]. In addition, the strain *C. cladosporioides* Hu01, which could represent an ancestral lineage respect to B1, has been described as degrader of the insecticide chlorpyrifos (50 mg L^−1^) given its hydrolysis product 3,5,6-trichloro-2-pyridinol (TCP) [38].

Several fungal species have been reported to degrade imidacloprid, with some strains achieving high degradation efficiencies. However, in all previously reported studies, the initial concentration of the active ingredient did not exceed 25 mg L^−1^ [17,18,19,20,21,39] (Table 4). In the present study, *C. cladosporioides* B1 tolerated and degraded substantially higher imidacloprid concentrations (20, 40, and 100 mg L^−1^), reaching final degradation efficiencies of 71%, 56%, and 30%, respectively. Although degradation efficiency decreased as the initial concentration increased, the strain maintained its biodegradation capacity even at 100 mg L^−1^, demonstrating a remarkable tolerance to elevated imidacloprid levels.

Although *Cladosporium* is generally considered a relatively slow-growing fungus compared with other filamentous ascomycetes, growth rate alone may not determine the suitability of a fungal strain for bioremediation. Other characteristics, such as tolerance to the contaminant, degradation efficiency, degradation kinetics, and transformation of the pollutant into less toxic products, should also be considered. In this regard, the comparison presented in Table 4 indicates that *C. cladosporioides* B1 degraded 71% of the initial imidacloprid concentration within 8 days, whereas *Trichoderma* and *Aspergillus*, which are generally considered faster growing fungi, required approximately 15 days to achieve similar degradation efficiencies (70–74%). Although these studies were performed under different experimental conditions, this comparison indicates that the relatively slow growth of *C. cladosporioides* does not necessarily translate into slow pesticide transformation. Therefore, *C. cladosporioides* B1 may have potential not only in the natural attenuation of pesticide-contaminated environments but also in controlled fungal-based treatment systems. Further studies using contaminated soils or agricultural effluents and larger-scale systems are needed to determine its practical applicability.

According to Randhawa [22], only a limited number of fungal strains capable of degrading neonicotinoid insecticides have been described to date. Most of these strains were isolated from environments such as wood chips [21], rotten wood [39], and agricultural wastewater [18]. Among the published studies, only Gangola et al. [17] reported fungal isolates obtained from pesticide-contaminated soil, and these strains degraded imidacloprid at concentrations of up to 20 mg L^−1^. Therefore, the identification of new fungal isolates with enhanced functional traits, including tolerance to high contaminant concentrations, high degradation efficiency, and rapid degradation kinetics, remains essential for the development of effective fungal-based technologies for the treatment of pesticide-contaminated agro-industrial effluents and biobed systems.

The results of the degradation kinetics showed higher degradation rates between days 2 and 8, corresponding to the exponential growth phase of *C. cladosporioides* B1. These results are in agreement with those reported by Mohammed and Badawy [18] and Zhu et al. [20] for *Aspergillus terreus* YESM3 and *Phanerochaete chrysosporium*, respectively, while other authors described longer kinetics with a higher degradation rate for *P. chrysosporium* between 7 and 21 days [21,39] (Table 4).

The initial concentration of imidacloprid markedly influenced the degradation kinetics of *C. cladosporioides* B1. The degradation rate increased significantly as the initial concentration was raised from 17.25 ± 0.04 to 42.86 ± 4.53 mg L^−1^ (Table 2), whereas no further increase was observed at 99.19 ± 13.46 mg L^−1^. The effect of substrate concentration on microbial biodegradation has been widely documented, with both very low and excessively high contaminant concentrations reducing degradation efficiency [40]. According to Zhu et al. [20], low substrate concentrations may limit enzyme–substrate interactions, whereas high concentrations can inhibit fungal metabolism because of the toxic effects of the parent compound and/or its transformation products. The plateau observed at the highest imidacloprid concentration suggests that *C. cladosporioides* B1 approached its physiological tolerance limit, resulting in no further increase in degradation rate despite the greater substrate availability.

Few studies have reported kinetic parameters for fungal degradation of imidacloprid, making direct comparisons difficult. Hu et al. [19] reported a degradation rate of only 0.049 mg L^−1^ d^−1^, considerably lower than the values obtained in the present study (1.42 ± 0.31–2.66 ± 0.75 mg L^−1^ d^−1^). Moreover, approximately 70% of the initial imidacloprid was degraded within eight days, demonstrating that *C. cladosporioides* B1 exhibits a substantially faster degradation capacity than previously reported fungal isolates.

The kinetic modeling further revealed that the inhibited Monod model described fungal growth much better than the classical Monod model at all tested imidacloprid concentrations. The presence of imidacloprid led to a significant increase in the growth rate compared to the control group without pesticide. This suggests that the fungus can use the compound or one of its metabolites as an additional source of carbon and/or nitrogen. However, as the initial concentration of the pesticide increased, the growth rate decreased slightly (0.49, 0.39, and 0.36 g L^−1^ d^−1^ for 20, 40, and 100 mg L^−1^, respectively), indicating that the initial stimulatory effect is gradually offset by toxic effects. Phugare et al. [35] and Mohamed and Badawy [18] reported the inhibition of microbial growth with increasing imidacloprid concentration. In addition, Zhu et al. [20] reported an increase in the viability of *Phanerochaete chrysosporium* during the first 24 h. However, it subsequently declined as imidacloprid concentration increased from 2 to 50 mg L^−1^.

Interestingly, although the specific growth rate (µ) decreased with increasing imidacloprid concentration, the degradation yield (Y_I/X_) increased. This indicates that less biomass was produced per unit of imidacloprid degraded, suggesting a partial uncoupling between fungal growth and pesticide degradation. Such behavior may reflect the allocation of metabolic energy toward cellular maintenance and stress response rather than biomass production. Alternatively, the existing fungal biomass may remain metabolically active and continue degrading imidacloprid despite reduced growth. Together with the temporal induction of laccase activity during the period of maximum degradation, these findings support a growth-associated degradation process in which extracellular oxidative metabolism plays a key role during imidacloprid transformation.

In contrast, the present results indicate that the transformation products generated by *C. cladosporioides* B1 did not markedly impair fungal growth. Instead, they appeared to reduce degradation efficiency at higher concentrations without causing substantial biomass loss, highlighting the remarkable tolerance of this strain to elevated imidacloprid levels.

The final pH values measured following degradation were lower than those of the control without the insecticide (by approximately one point). These results are consistent with those reported for the degradation of imidacloprid by *T. versicolor*. According to the study by Hu et al. [19], the decrease in pH was caused by acid secretion from the fungi. Changes in pH are directly related to the transformation products and the degradation pathway.

Further assays varying the type of carbon source and its concentration, the amount of inoculum, and the pH, among other factors, should be carried out to optimize the degradation process by *C. cladosporioides* B1.

Laccase enzymes are considered ‘green’ catalysts because they use O_2_ as a co-substrate and produce H_2_O as a by-product. These enzymes catalyze the oxidation of various types of phenols and similar aromatic compounds such as aromatic amines [32,41,42]. In this study, laccase activity was induced during the course of degradation tests in batch cultures, reaching its highest values during the period of the maximum imidacloprid degradation rate, and a strong correlation with this was observed, indicating the potential involvement of laccase in the degradation pathway of imidacloprid by *C. cladosporioides* B1. As shown in Table 4, there are no reports of laccase activity directly involved in imidacloprid degradation by fungi.

Although the fungal breakdown of NEOs has been described as primarily carried out by internal enzymes such as cytochrome P450 and peroxidase (POD), it is also carried out by extracellular enzymes like lignin peroxidase, manganese peroxidase, laccase, and apoenzymes produced by fungal mycelia [22]. As reported by Weaber et al. [21], significant degradation of imidacloprid was observed in the presence of the cytochrome P450 enzyme inhibitor 1-amino-benzotriazole (ABT) for *Phanerochaete chrysosporium*. Indeed, treatments containing the enzyme inhibitor demonstrated comparable outcomes to those devoid of the inhibitor. Furthermore, the study by Mori et al. [39] found that 1-ABT moderately inhibited imidacloprid degradation for *P. chrysosporium*. The results suggest that the degradation of imidacloprid may be the result of more than one enzyme or indeed a combination of enzymes. The findings presented herein demonstrate that laccase activity does not increase proportionately with imidacloprid concentration. If laccase were the sole limiting factor in degradation, one would expect a much greater enzymatic response at 100 mg L^−1^. Instead, what is observed is primarily a temporal shift in the peak activity. This pattern could indicate that laccase activity may contribute to the degradation process, but it would not necessarily account for the observed degradation at higher imidacloprid concentrations. Other stress-induced metabolic responses and/or the contribution of additional enzymes could also be involved. Further studies would be required to elucidate the relative contribution of laccase and other enzymatic and metabolic mechanisms to imidacloprid degradation by *C. cladosporioides*.

As Weaber et al. [21] mentioned, identifying transformation products is essential for determining whether the degradation process could cause additional environmental damage. In this study, some metabolites resulting from fungal degradation were identified, and their toxicity was evaluated. The degradation of commercial-quality imidacloprid produced 5-hydroxyimidacloprid, imidacloprid olefin, and 6-chloronicotinic acid. Dai et al. [43] reported that imidacloprid olefin is significantly more harmful than imidacloprid itself. Nevertheless, this study revealed that the degradation products of commercial imidacloprid were not harmful to earthworms, unlike undegraded imidacloprid. In addition, 6-chloronicotinic acid was detected as a degradation product, suggesting that imidacloprid olefin could transform into 6-chloronicotinic acid (Scheme 2), as Kathuria et al. [14], Guo et al. [44], and Zhu et al. [20] also showed.

Most studies on NEOs have focused on their toxicity to pollinators and aquatic insects, neglecting their effects on soil invertebrates [45]. Among the latter, several earthworm species (e.g., *E. fetida* and *E. andrei*) have occupied an important place in toxicity testing [46], as they are standard bioindicators representative of the soil community. However, as previously stated, the avoidance behavior exhibited by earthworms (*E. fetida*) toward imidacloprid (EC50 0.77 mg Kg^−1^) was found to be a more sensitive indicator of soil contamination with NEOs in comparison to acute toxicity [23], and the avoidance behavior is regarded as the method that provides first-hand information about the behavior of an organism in the presence of contaminants in the soil [47]. For this reason, an avoidance test was conducted in this study, and the results demonstrated that the degradation products generated by *C. cladosporioides* B1 possess a reduced toxic profile in comparison to imidacloprid. Conversely, *E. fetida* was attracted to the imidacloprid degradation product. This concurred with the presence of 6-chloronicotinic acid, which was reported as a less neurologically toxic metabolite from imidacloprid degradation [20].

Finally, the present study provides an integrated characterization of imidacloprid removal by the native strain *Cladosporium cladosporioides* B1, combining degradation kinetics at different pesticide concentrations, laccase activity, identification of transformation products, and assessment of the residual toxicity. This approach provides a broader evaluation of the biodegradation process and its environmental relevance, while contributing new information on the potential of a native fungal strain for imidacloprid transformation.

## 5. Conclusions

The *C. cladosporioides* B1 strain has a potent ability to transform imidacloprid into less toxic compounds. The fungus could tolerate and degrade initial imidacloprid concentrations ranging from 20 to 100 mg L^−1^. The maximum degradation (71%) was achieved with an initial concentration of 20 mg L^−1^ of imidacloprid; however, the maximum degradation rate was achieved with 40 mg L^−1^ (rate 2.66 mg L^−1^ d^−1^).

The results presented here support a growth-associated degradation process in which laccase induction accompanies the period of highest imidacloprid transformation, suggesting that fungal growth and extracellular oxidative metabolism are closely linked during biodegradation.

The transformation products showed lower toxicity in attraction tests against *E. fetida.* These characteristics make this strain an interesting alternative for treating agricultural liquid waste containing imidacloprid and for possible implementation in bioaugmenting biological beds or biofilters. Future research should focus on identifying the optimal conditions to increase the degradation rate, and the effectiveness of incorporating the B1 strain into biofilter or biological bed systems should be evaluated.

## Figures and Tables

**Scheme 1:**
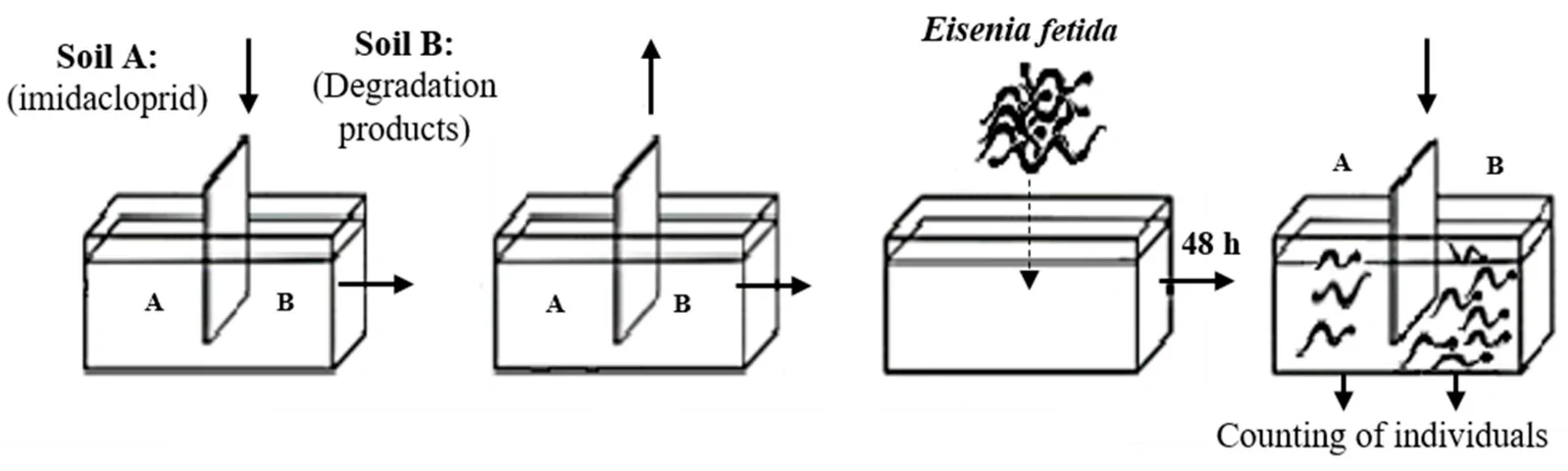
Avoidance behavior assay. A positive NR indicates avoidance of the treated soil, whereas 0% or a negative value indicates a non-response or attraction to the pesticide tested, respectively. To test significant differences between mean values, an ANOVA test was performed (*p* = 0.05).

**Figure 1:**
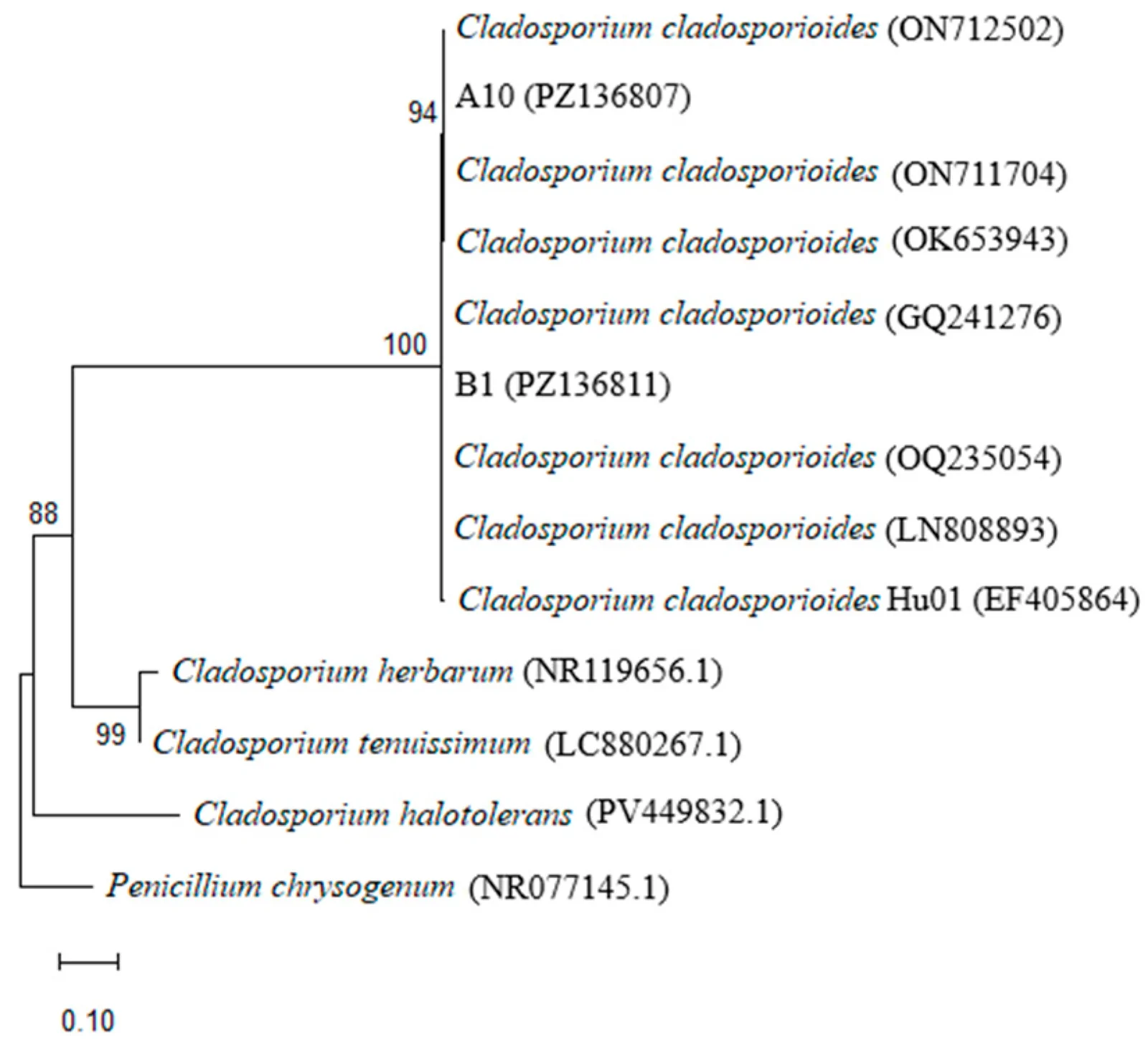
Phylogenetic tree of imidacloprid-degrading strains B1 and A10. Phylogenetic tree based on sequences from the ITS region of the rDNA of *Cladosporium* strains isolated from soil sourced from commercial strawberry crops that had previously been treated with imidacloprid. The numbers on the branches indicate the bootstrap values.

**Figure 2:**
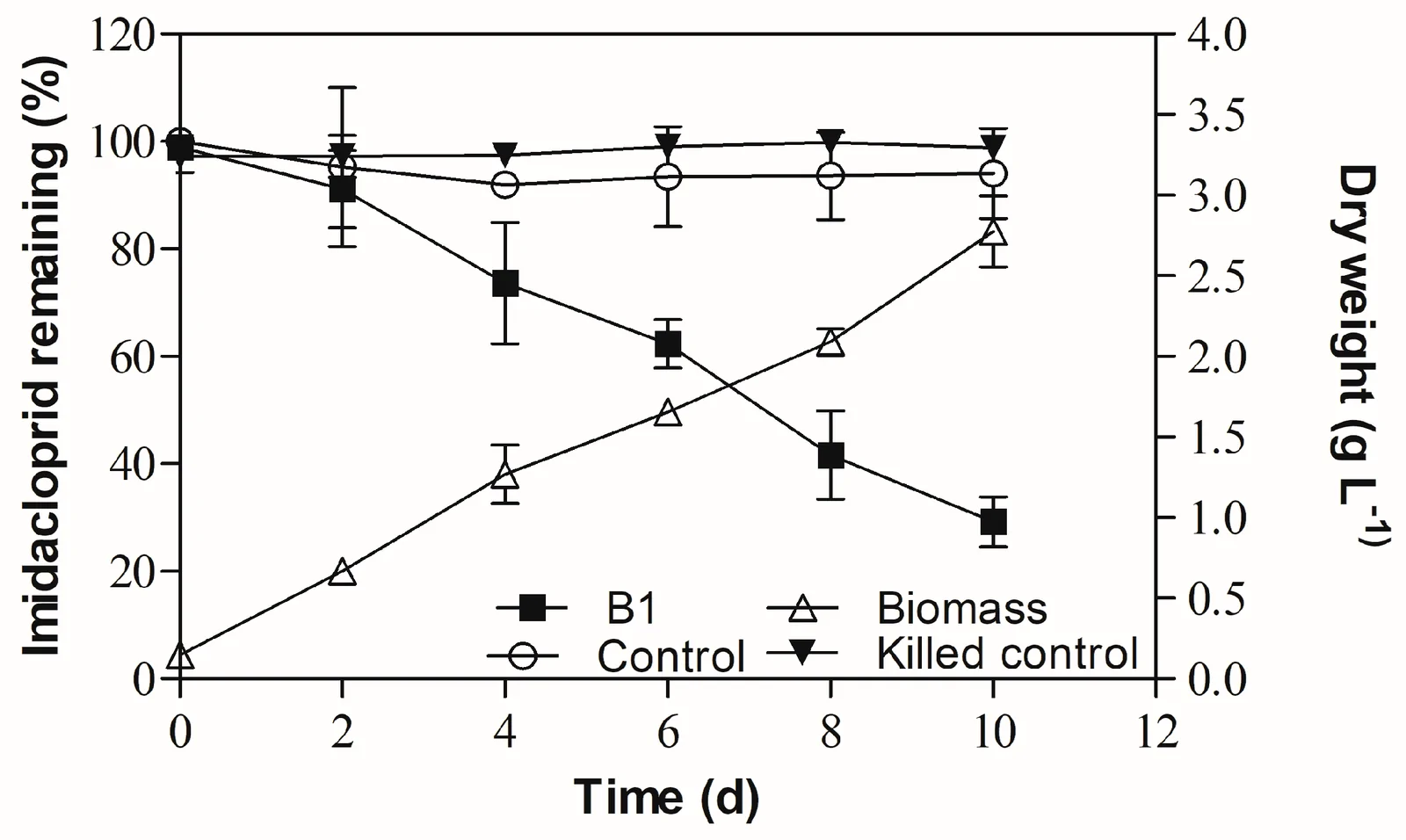
Degradation and growth kinetics in presence of imidacloprid by *C. cladosporioides* B1. LMS was supplemented with 10 g L^−1^ of glucose and 20 mg L^−1^ of imidacloprid. Control means LMS + imidacloprid without inoculation and killed control means adsorption control. Each point represents the mean value ± SD of three independent experiments.

**Figure 3:**
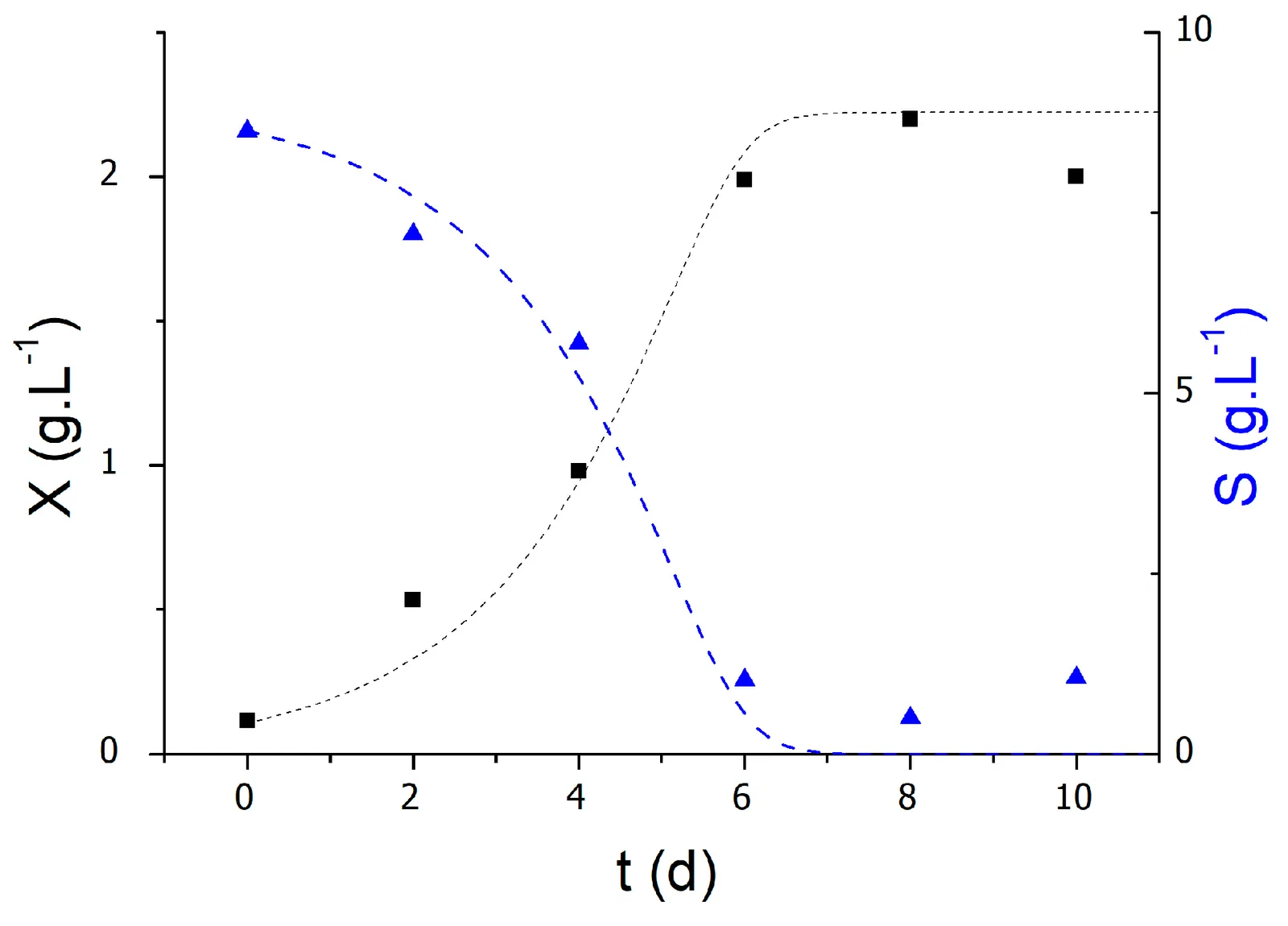
Growth kinetic and predictions of the Monod kinetic model. X: Biomass; S: Glucose. Experimental (symbols) and modeled data (dashed lines) in absence of imidacloprid.

**Figure 4:**
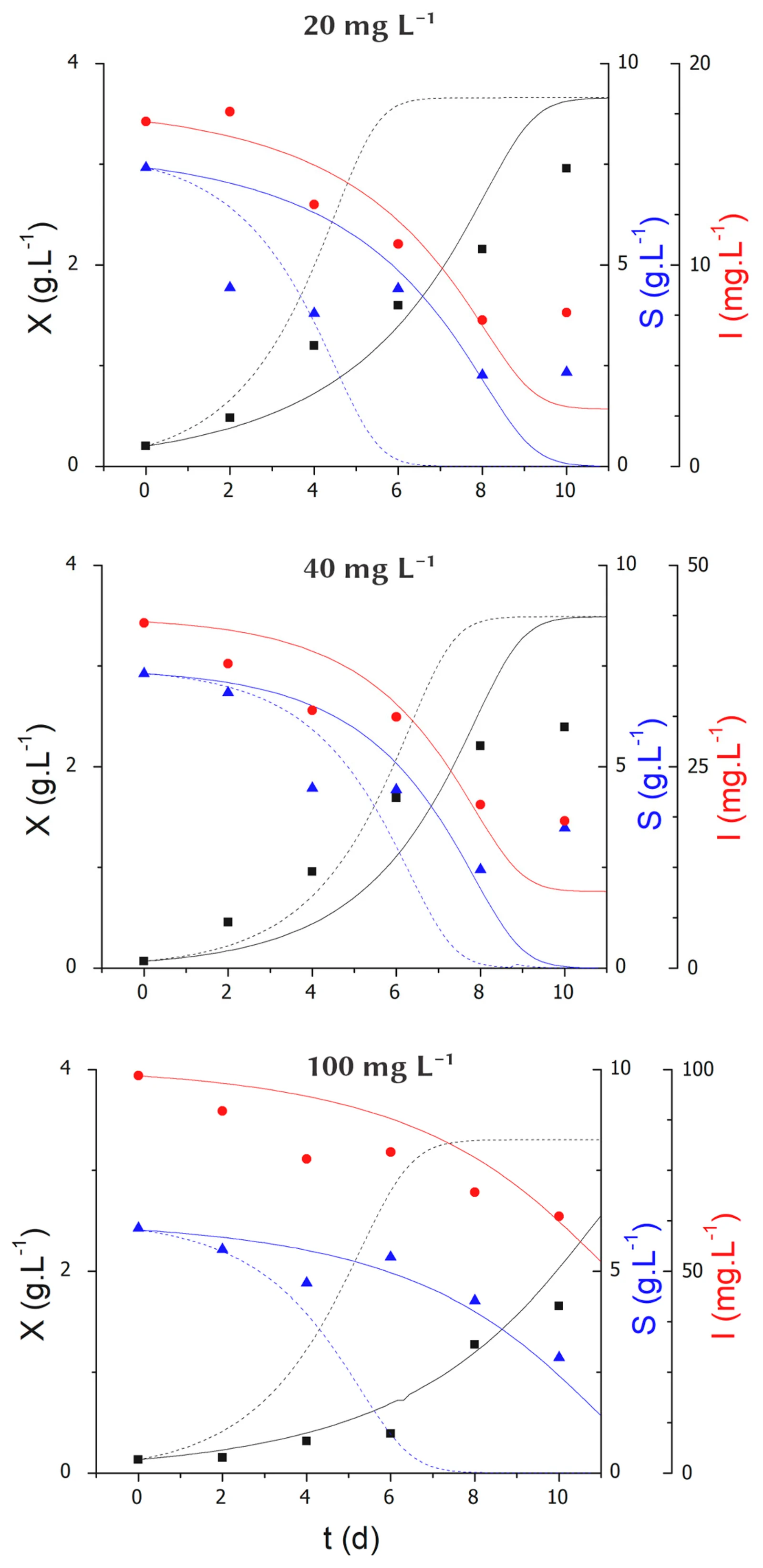
Degradation and growth kinetics by *C. cladosporioides* B1 at different initial concentrations of imidacloprid. X: Biomass; S: Glucose; I: Imidacloprid concentration. Experimental (symbols) and modeled data with the Monod kinetic model (dashed lines) and the inhibited Monod kinetic model (solid lines).

**Figure 5:**
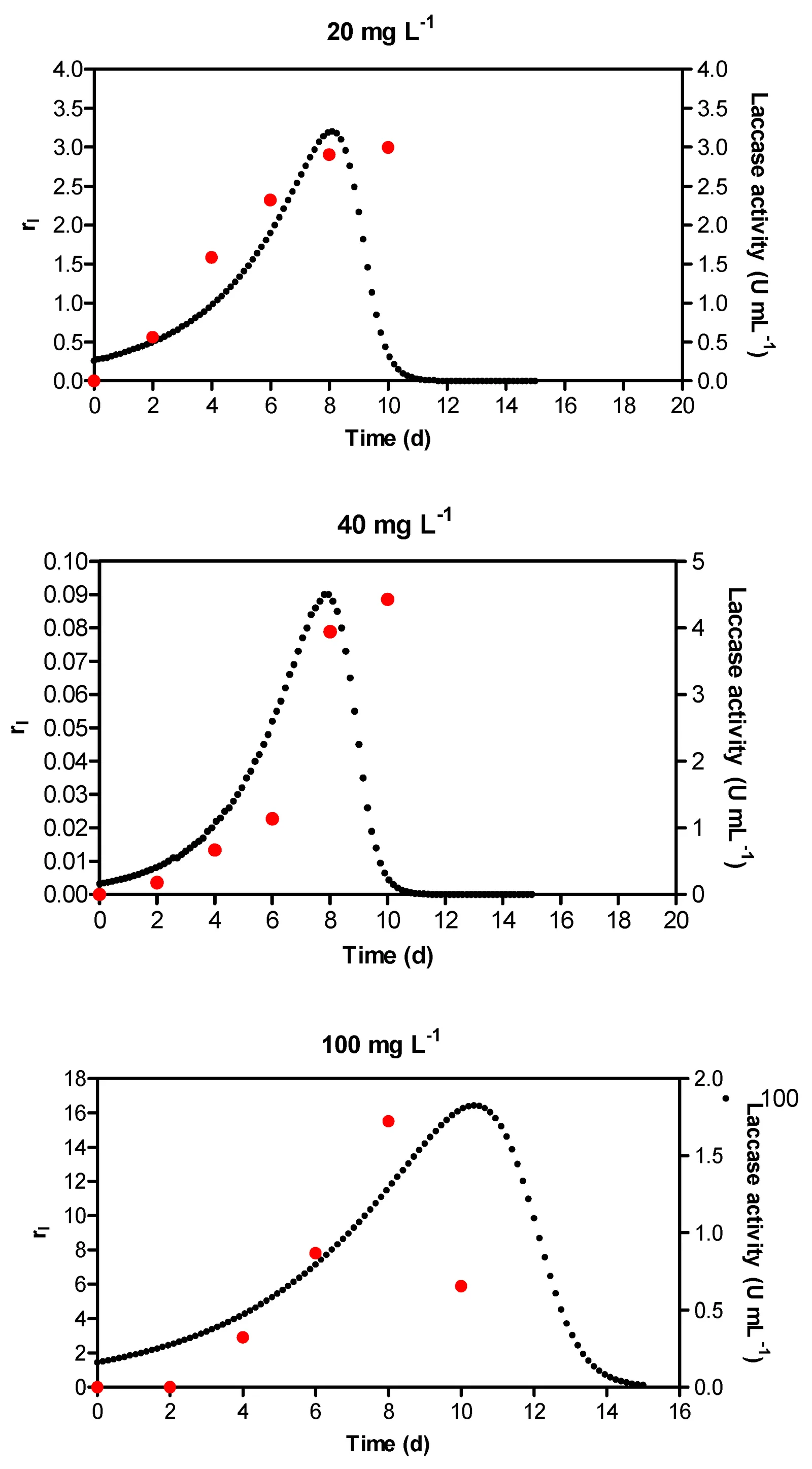
Time course of laccase activity and correlation with the imidacloprid degradation rate. Red circles indicate experimental laccase activity, and black circles indicate degradation rate (r_I_). Each experimental point represents the mean value ± SD of three independent experiments.

**Figure 6:**
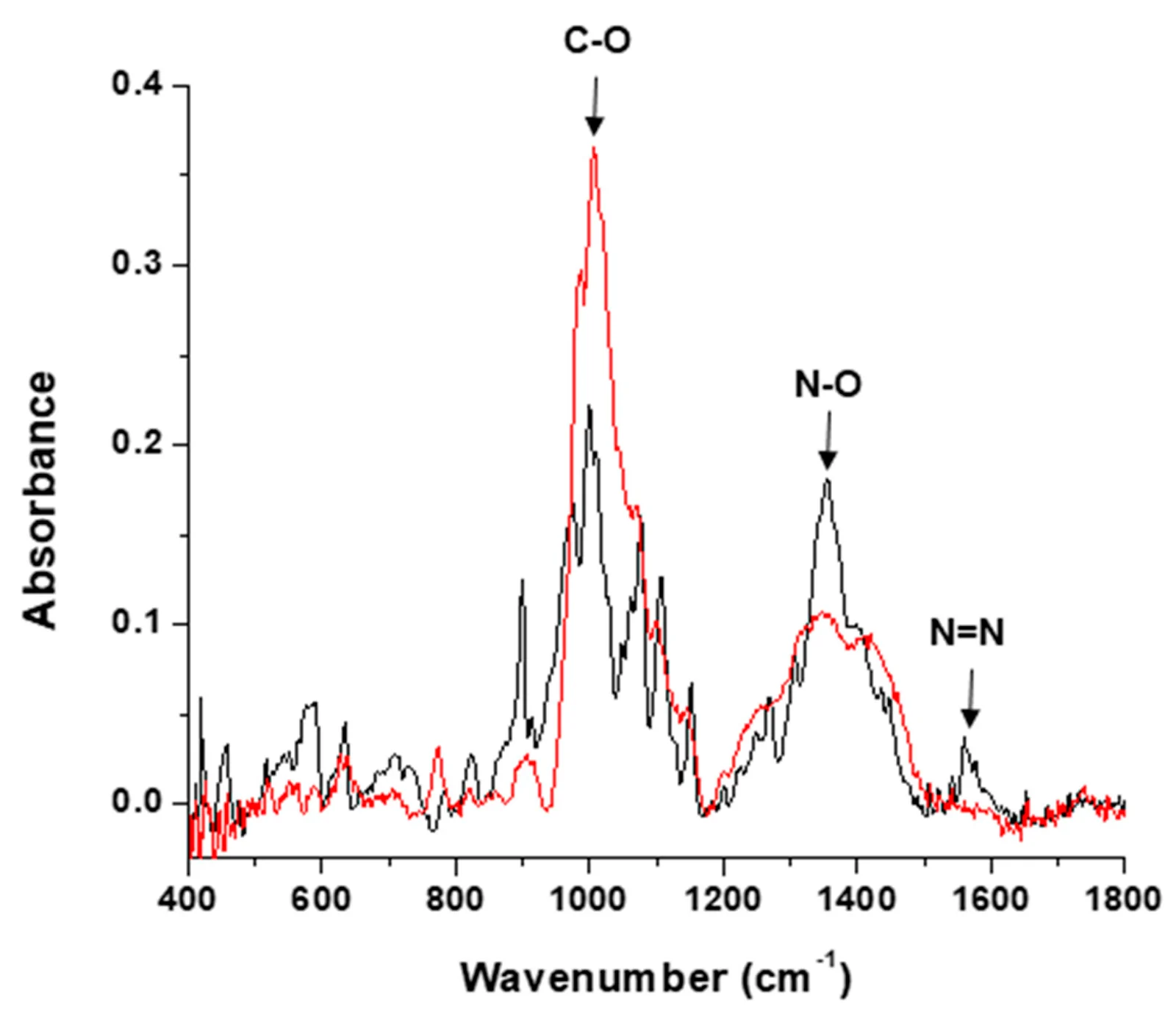
FTIR spectra of imidacloprid and its degradation products after *C. cladosporioides* B1 degradation. Degradation of high-purity (≥98%) analytical standard of imidacloprid, control standard imidacloprid (black line), and degradation products (red line).

**Figure 7:**
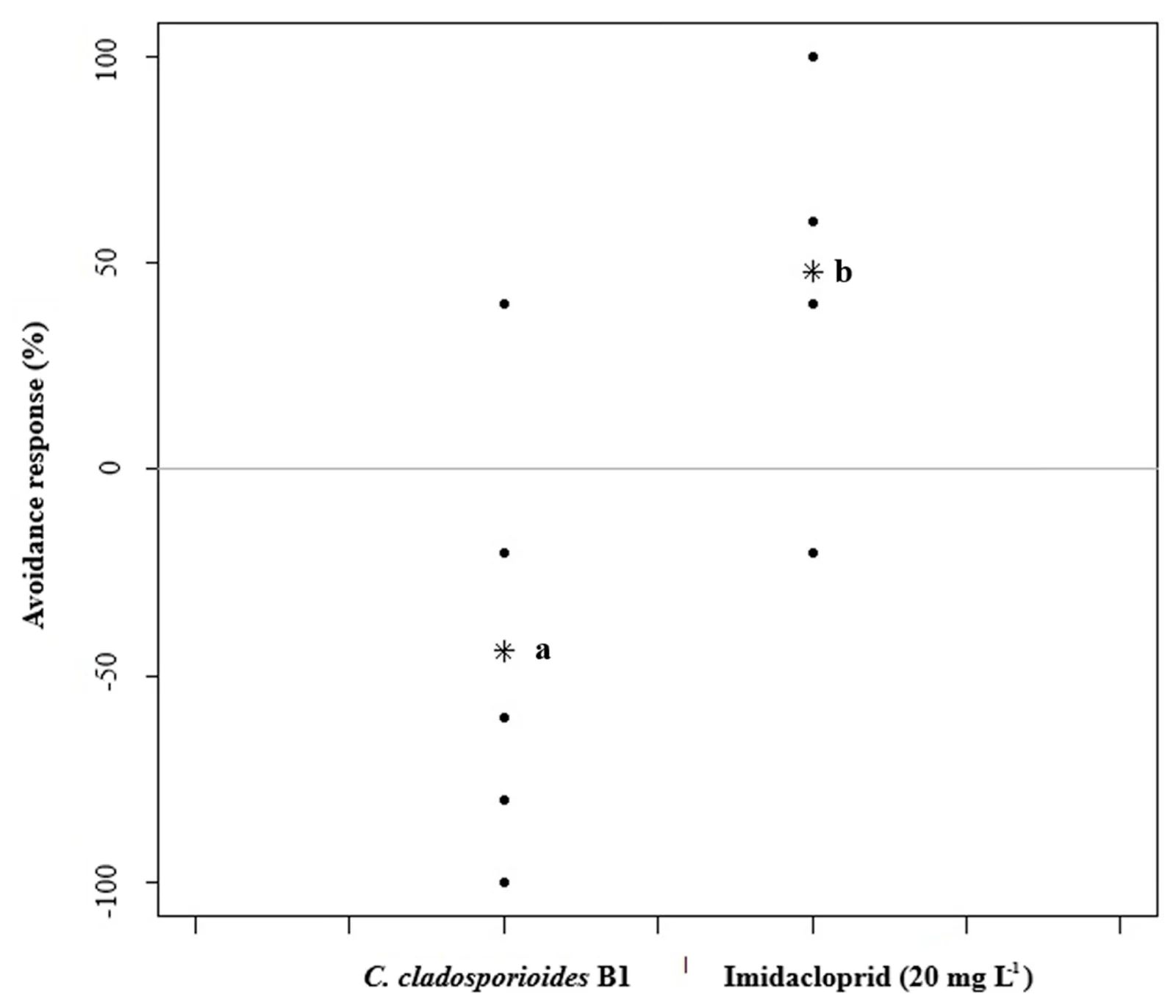
Avoidance response (%) of *E. fetida* when exposed to imidacloprid (20 mg L^−1^) versus degradation products after 48 h. The asterisks represent the average values and the circles represent the replicates. Different letters mean significant differences at *p* < 0.05 (ANOVA test).

**Scheme 2:**
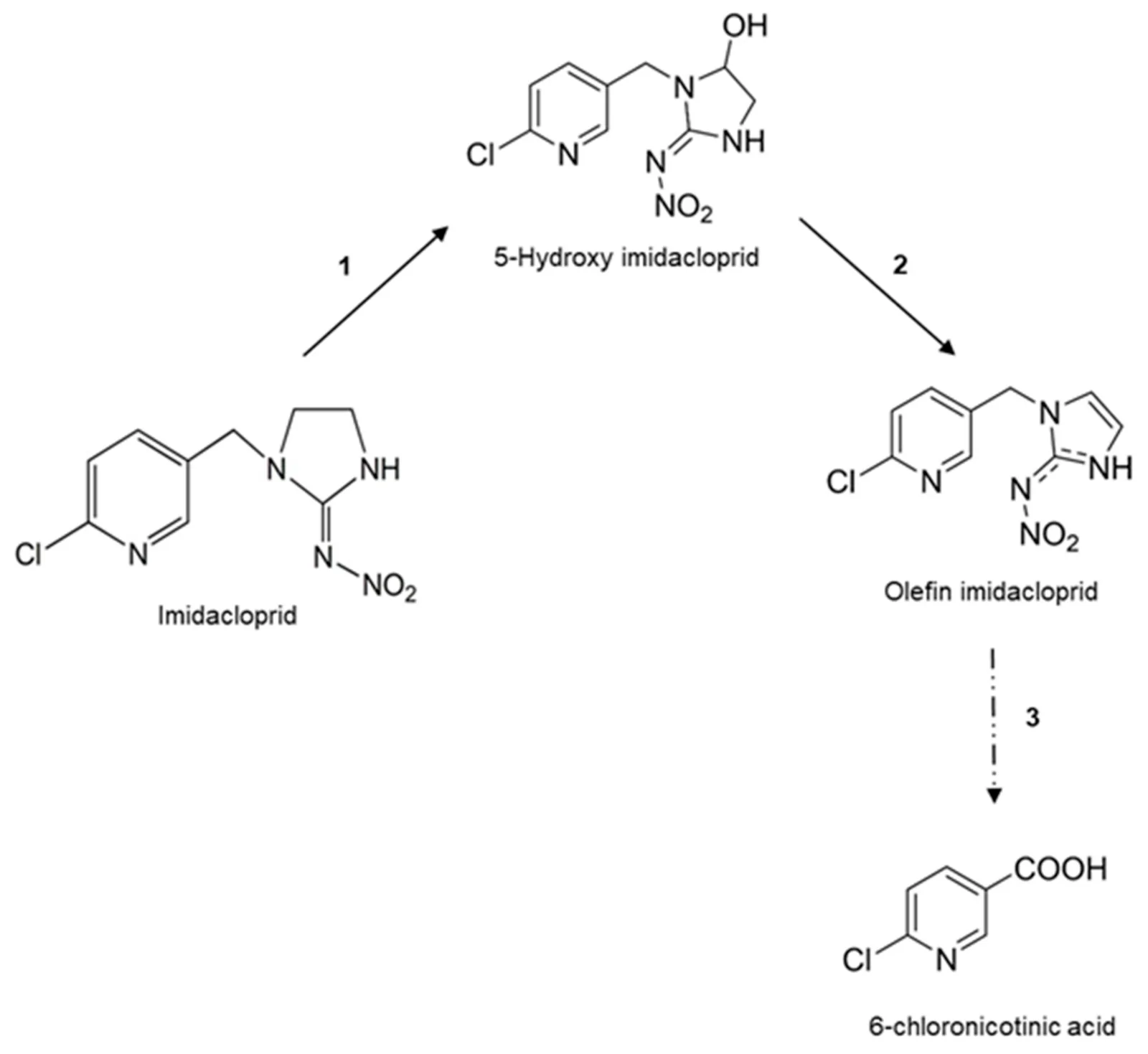
Proposed biotransformation pathway of imidacloprid by *C. cladosporioides* B1. 1: Hydroxylation, 2: Elimination by laccase activity, 3: Dashed arrows indicate reactions steps to be confirmed.

**Table 1:** NCBI Blast sequencing results of the isolated strains.

Strain	Gender and Species	ID (%)	Coverage (%)	Accession Number
LB_A2	*Apiospora esporlensis*	99 (MT856406)	95	PZ136801
LB_A3	*Cladosporium* sp.	100 (MK460326)	95	PZ136802
LB_A4	*Alternaria alternata*	100 (MN495846)	100	PZ136803
LB_A5	*Cladosporium halotolerans*	99 (OM349546)	100	PZ136804
LB_ A6	*Trichoderma koningiopsis*	100 (MK459337)	100	PZ136805
LB_A7	*Alternaria alternata*	100 (OQ371359)	100	PZ136806
LB_A10	*Cladosporium cladosporioides*	100 (ON712271.1)	83	PZ136807
LB_A11	*Trichoderma* sp.	100 (LC109302)	100	PZ136808
LB_A12	*Cladosporium cladosporioides*	100 (HM037955)	77	PZ136809
LB_Hm	*Epicoccum nigrum*	99 (MF435074)	95	PZ136810
LB_B1	*Cladosporium cladospoiroides*	100 (GQ241276)	100	PZ136811
LB_B2	*Fusarium merismoides*	97 (MW221144)	67	PZ136812
LB_B3	*Trichoderma velotinum*	94 (MH651379)	100	PZ136813

**Table 2:** Kinetic data and model parameters.

Imidacloprid Initial Concentration (mg L^−1^)	Degradation (%) *	Initial Degradation Rate (mg L^−1^ d^−1^)	Growth Rate (g L^−1^d^−1^)	μmax (d^−1^)	K (g L^−1^)	Y_X/S_ (g_X_ g_S_^−1^)	K_I_ (mg L^−1^)	Y_I/X_ (mg_I_ g_X_^−1^)
0		--	0.17 ± 0.05 ^a^	0.76 ± 0.04	1.96 ± 0.24	0.25 ± 0.001	--	--
17.25 ± 0.04	71.43 ± 3.49 ^a^	1.42 ± 0.31 ^a^	0.49 ± 0.06 ^b^	“	“	0.47 ± 0.13	19.0 ± 1.85	4.13 ± 0.38
42.86 ± 4.53	56.37 ± 11.71 ^a^	2.66 ± 0.75 ^b^	0.39 ± 0.04 ^b^	“	“	0.47 ± 0.06	150.0 ± 19.50	9.79 ± 0.60
99.19 ± 13.46	30.28 ± 9.64 ^b^	2.47 ± 0.93 ^ab^	0.36 ± 0.02 ^b^	“	“	0.53 ± 0.07	89.1 ± 7.70	17.42 ± 3.86

* Degradation percentage reported corresponds to day 10 of the kinetics. Data are expressed as the means of three independent experiments ± SD. Different letters mean significant differences at *p* < 0.05 (Kruskal-Wallis test).“ Means same value.

**Table 3:** LC-DAD and LC-MS/MS operating parameters for imidacloprid and its degradation products.

Analyte	Molecular Mass	RT (Time)	Ionization Mode	Q Transition	q Transition
Imidacloprid	256	8.3			
5-Hydroxy imidacloprid	272	2.35	+	272 > 225	272 > 191
Imidacloprid olefin	251.9	2.3	+/−	254 > 205	254 > 171
6-Chloronicotinc acid	156.1	2.57	+/−	158.2 > 122.1	158.2 > 112.1

LC-DAD: Liquid chromatography with diode array detector; LC-MS/MS: Liquid chromatography–tandem mass spectrometry; RT (min): Retention time; Q transition: Transition of the major ion product; q transition: Transition of the secondary ion product.

**Table 4:** Degradation studies of imidacloprid by fungi.

Microorganisms	Isolated Media	Initial Concentration (mg L^−1^)	Time (Days)	Degradation Percentage (%)	Metabolites	Enzymes Involved	References
*Aspergillus oryzae*	Soil	20	15	74	-	-	[17]
*Aspergillus terreus* YESM3	Agricultural wastewater	25	20	96	6-Chloronicotinc acid	-	[18]
*Phanerochaete chrysosporium*	Culture collection	0.5	28	90	Imidacloprid nitrosoguanidine Imidacloprid olefin 6-Chloro-3 pyridylmethanol	-	[21]
		20	6	67	6-Chloronicotinc acid 5-Hydroxy-imidacloprid Imidacloprid- olefin	LiP, MnP, ROS	[20]
		25	28	97	6-Chloro-3-piridinemetanol	Cytochrome P450	[39]
*Trametes versicolor*	Culture collection	4	7	65	5-Hydroxy-imidacloprid 6-Chloronicotinc acid	Cytochrome P450	[19]
*Trichoderma longibrachiatum*	Soil	20	15	70	-	-	[17]
*Cladosporium cladosporioides*	Soil	20 40 100	8	71 56 30	6-Chloronicotinc acid 5-Hydroxy-imidacloprid Imidacloprid- olefin	Laccase	Present study

## Data Availability

All data are available in the Repositorio Institucional CONICET Digital: http://hdl.handle.net/11336/270846 (accessed on 17 August 2026).

## Supplementary Materials

The following supporting information can be downloaded at: https://www.mdpi.com/article/10.3390/jof12100724/s1, Supplementary Material Transformation products of imidacloprid in the pure standard and commercial formulation treated with or without *Cladosporium cladosporioides* B1.

## Abbreviations

The following abbreviations are used in this manuscript:

ITS	Internal transcribed spacer
LCMS/MSMS	Liquid chromatography–tandem mass spectrometry
AT-FTIR	Attenuated total reflectance–Fourier transform infrared
RT	Retention time
NEOs	Neonicotinoid insecticides
IS	Internal standard
PTFE	Polytetrafluoroethylene
ABTS	2,2′-Azino-bis(3-ethylbenzothiazoline)-6-sulfonic acid
PDA	Potato dextrose agar
LMS	Minimal mineral salt medium
LC-DAD	Liquid chromatography with diode array detector
NR	Net response
PHAs	Polyhydroxyalkanoates
TCP	3,5,6-Trichloro-2-pyridinol
POD	Peroxidase
