# Supplementary material for: First Report of Imidacloprid Biodegradation by Cladosporium cladosporioides: Kinetic Modeling, Transformation Products, and Toxicity Reduction
Source: J Fungi (Basel). 2026 Sep 27;12(10):724. doi: 10.3390/jof12100724 (PMC13705660; doi:10.3390/jof12100724)

SUPPLEMENTARY MATERIAL

First report of imidacloprid biodegradation by *Cladosporium cladosporioides*: kinetic modelling, transformation products and toxicity reduction

Melissa C. Aguirralde[1,3], Cintia A. Mazzucotelli[1], Ignacio Durruty[1,2], Débora Nercessian[2,5], Débora J. Pérez[2,6], Carla Salvio[6], Marcelo C Sosa Morales[4] and Erika A Wolski[1,2]*

1 Instituto de Ciencia y Tecnología en Alimentos y Ambiente (INCITAA), Facultad de Ingeniería, Universidad Nacional de Mar del Plata, J.B. Justo 4302, 7600 Mar del Plata, Buenos Aires, Argentina

2 Consejo Nacional de Investigaciones Científicas y Técnicas (CONICET), CCT-Mar del Plata, 7600 Mar del Plata, Buenos Aires, Argentina

3 Comisión de Investigaciones Científicas (CIC). Ministerio de Ciencia, Tecnología e Innovación de Buenos Aires, Mar del Plata, Argentina

4 Instituto de Investigaciones en Ciencia y Tecnología de Materiales (INTEMA)-CONICET. Facultad de Ingeniería, Universidad Nacional de Mar del Plata, Mar del Plata, Buenos Aires, Argentina

5 Instituto de Investigaciones Biológicas (IIB), CONICET, Universidad Nacional de Mar del Plata. Funes 3250 4to Nivel, 7600 Mar del Plata. Argentina.

6 Facultad de Ciencias Agrarias, Universidad Nacional de Mar del Plata. Ruta Nacional N° 226, Km 73.5, 7620 Balcarce, Argentina

* Correspondence: ewolski@mdp.edu.ar / erikawolski@gmail.com

Transformation products of imidacloprid in the pure standard and commercial formulation treated with or without *Cladosporium cladosporioides B1*

1. **Imidacloprid (Standard quality) without fungal treatment**

   - **6-chloronicotinic acid**

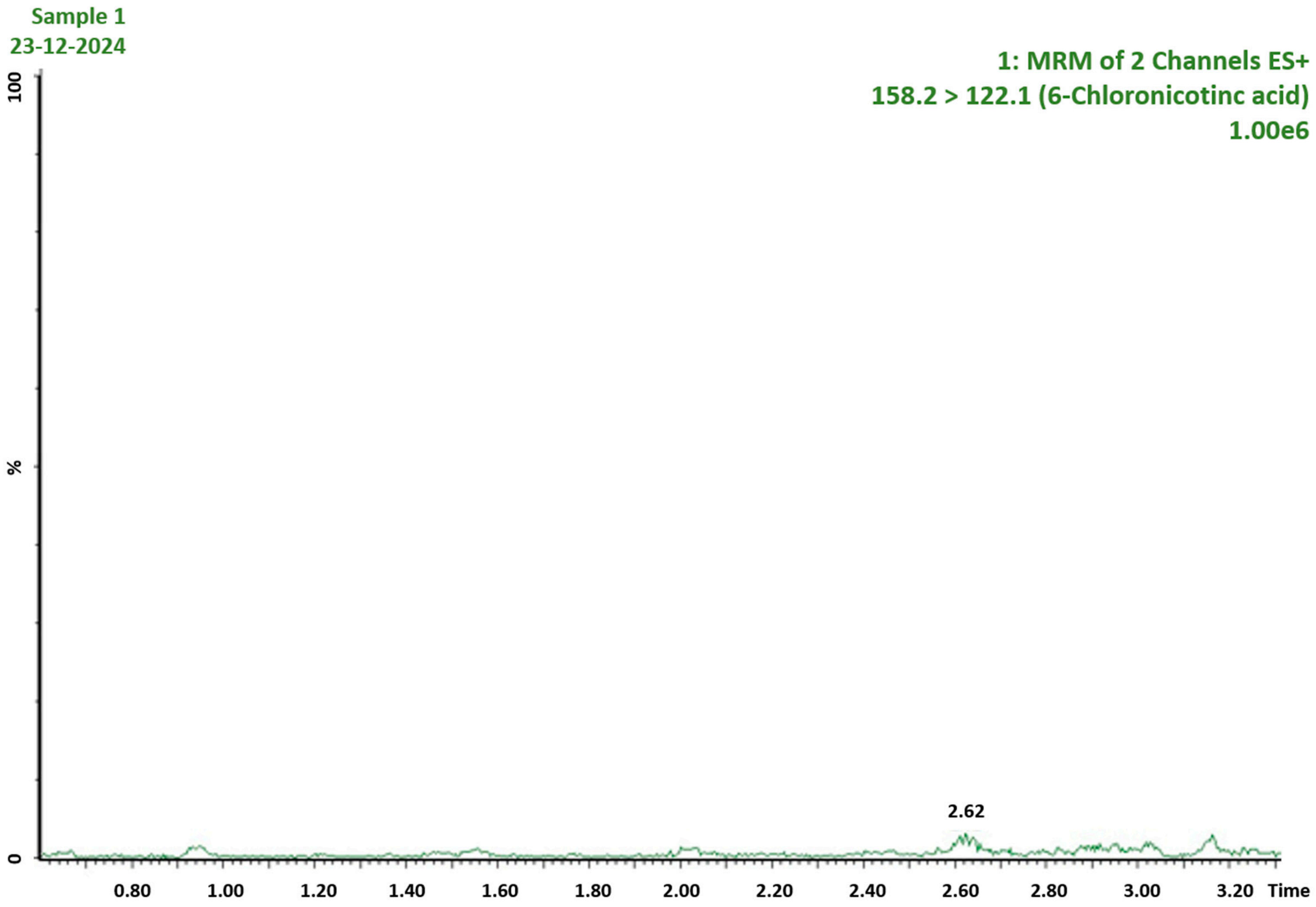

- **Imidacloprid-olefin**

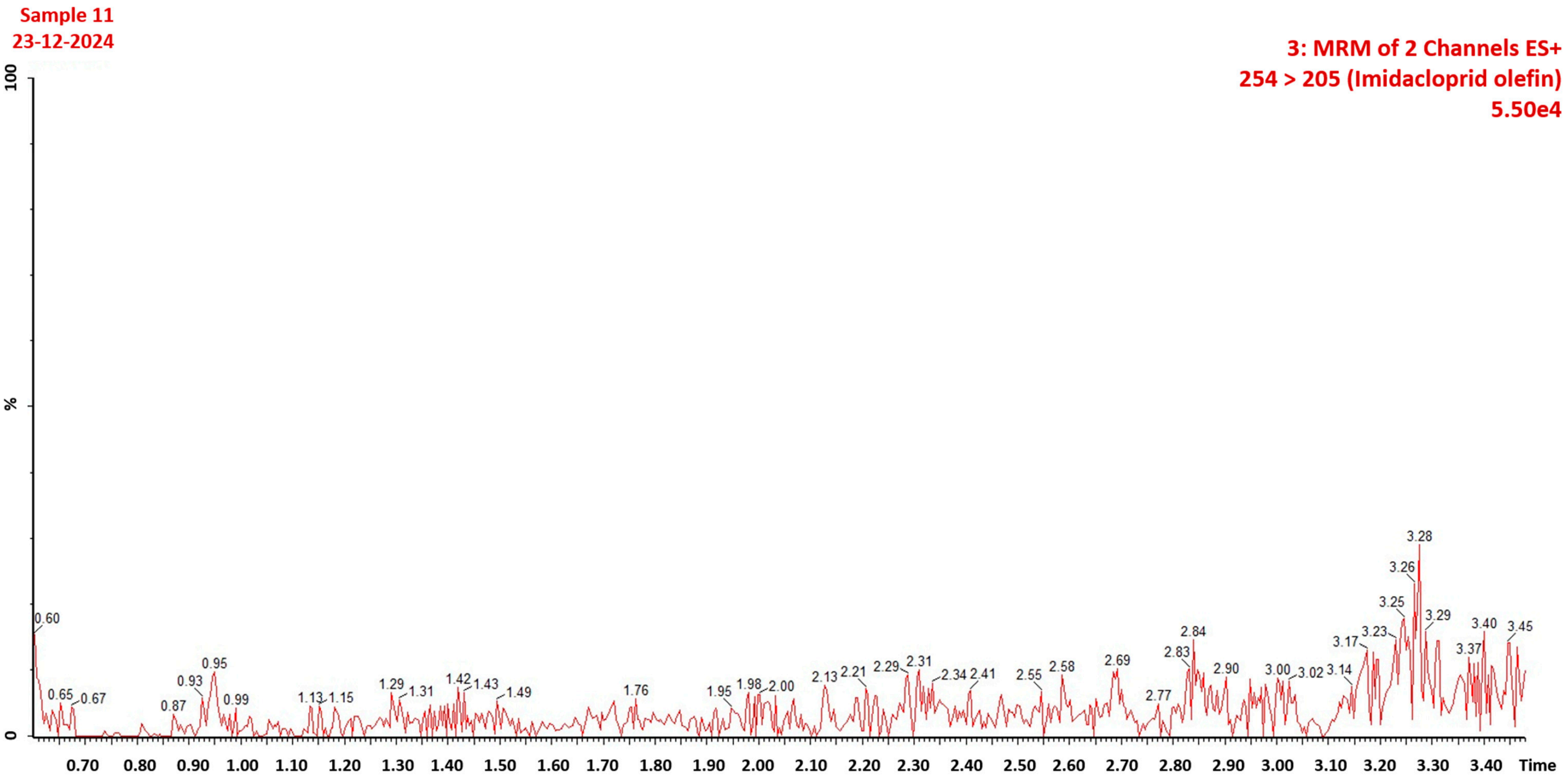

- **5-hydroxy imidacloprid**

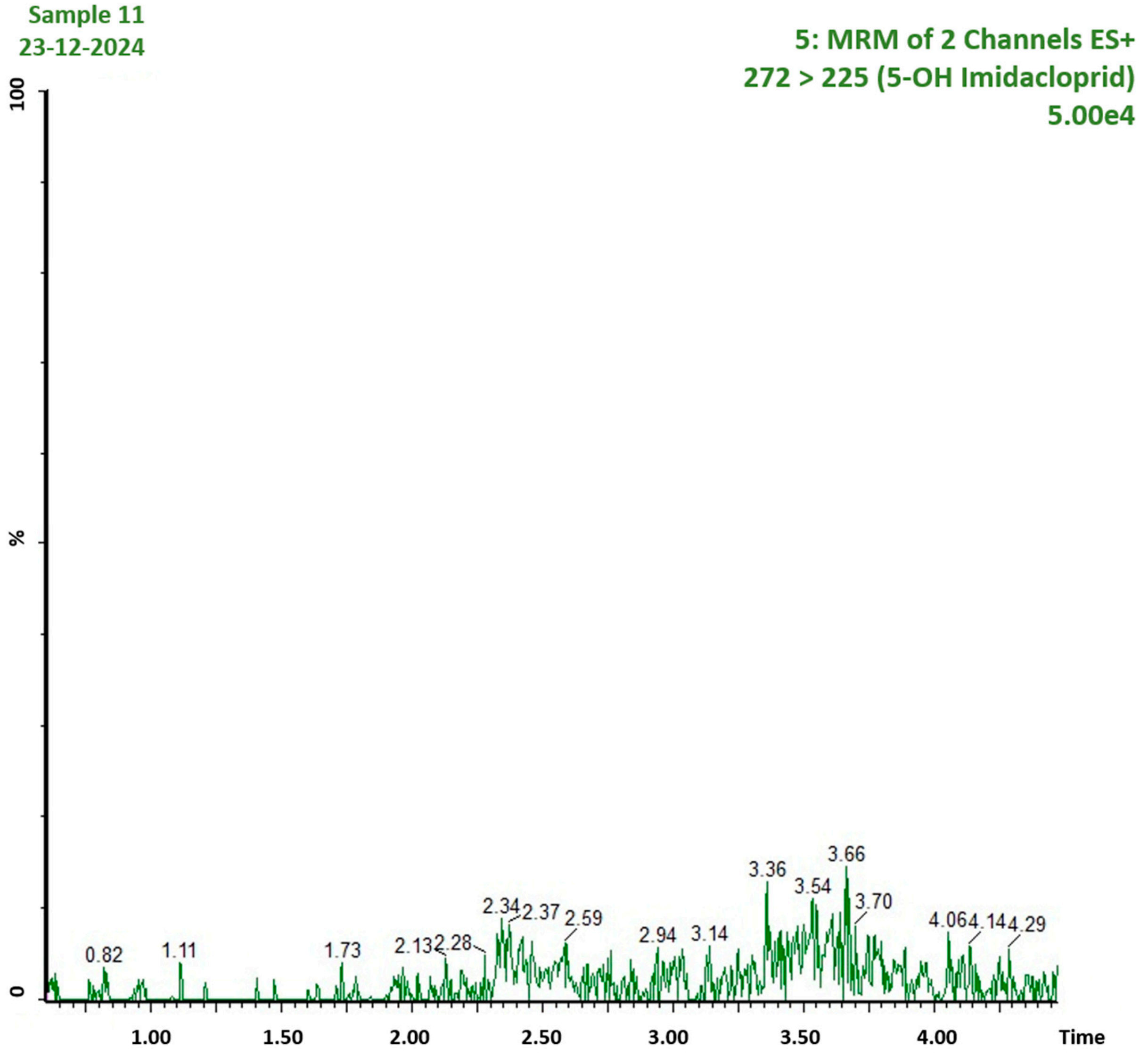

2. Imidacloprid (Standard quality) after fungal treatment

- 6-chloronicotinic acid

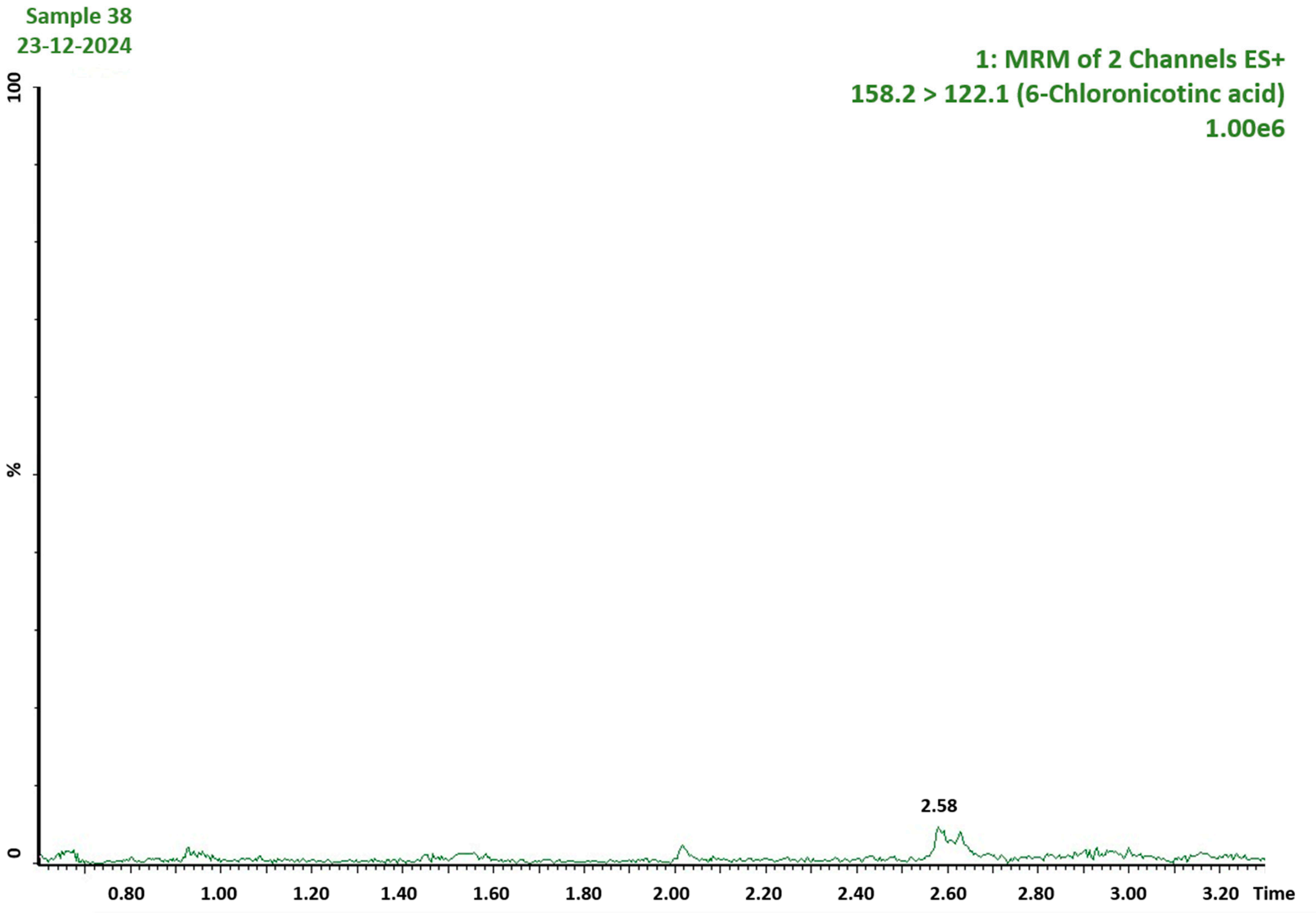

- Imidacloprid-olefin

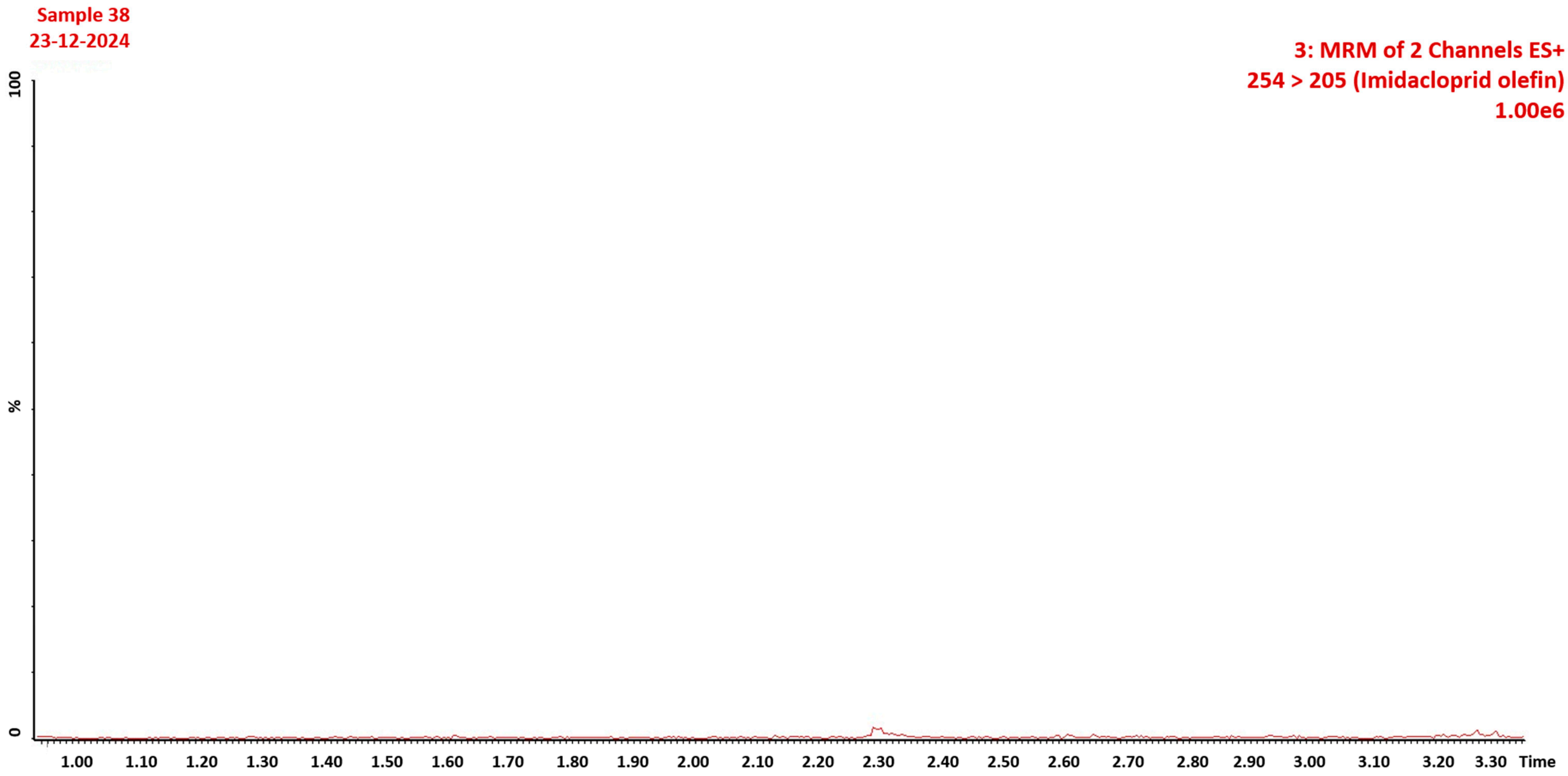

- **5-hydroxy Imidacloprid**

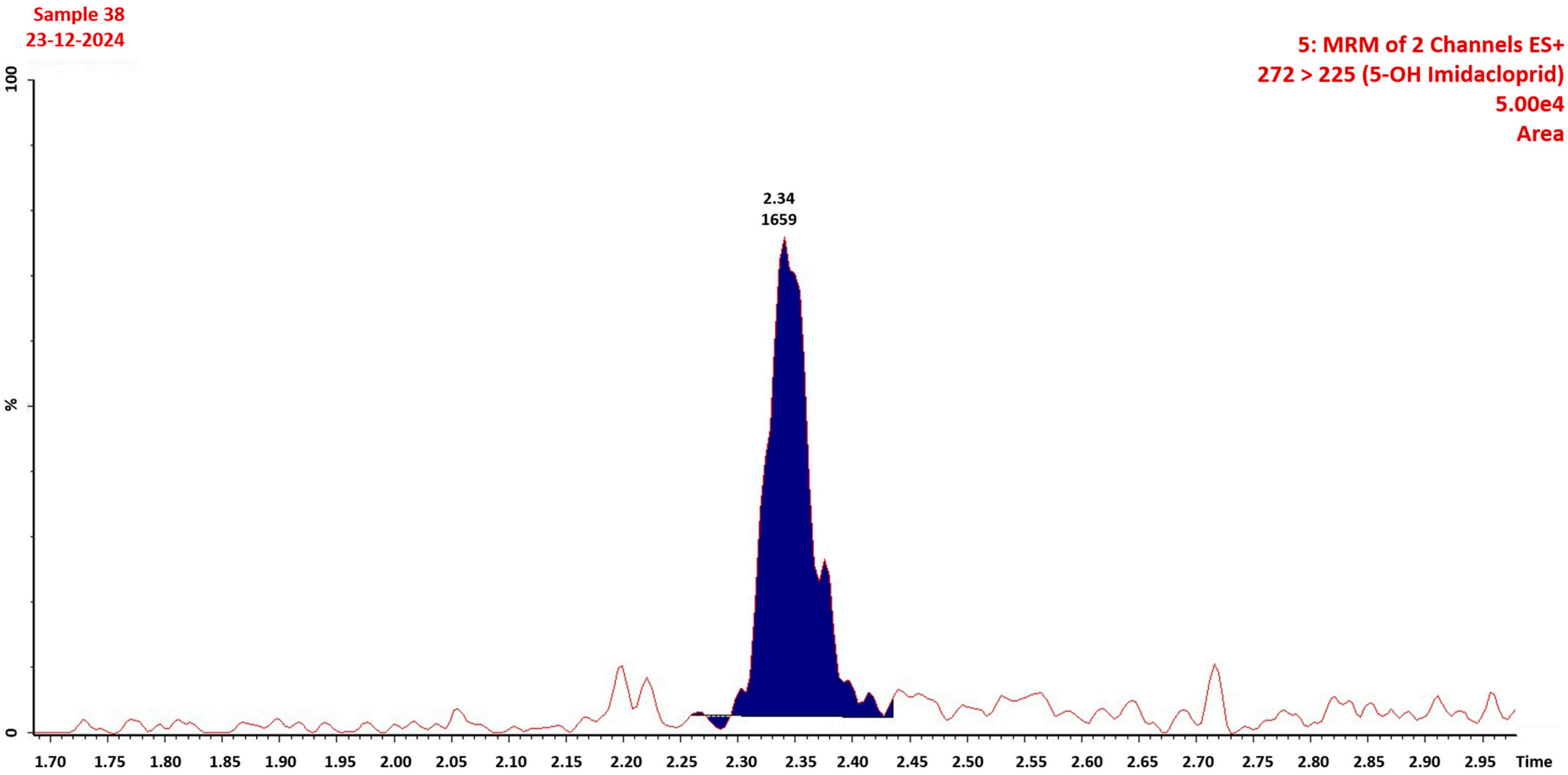

3. **Imidacloprid (Commercial quality) without fungal treatment**

- 6-chloronicotinic acid

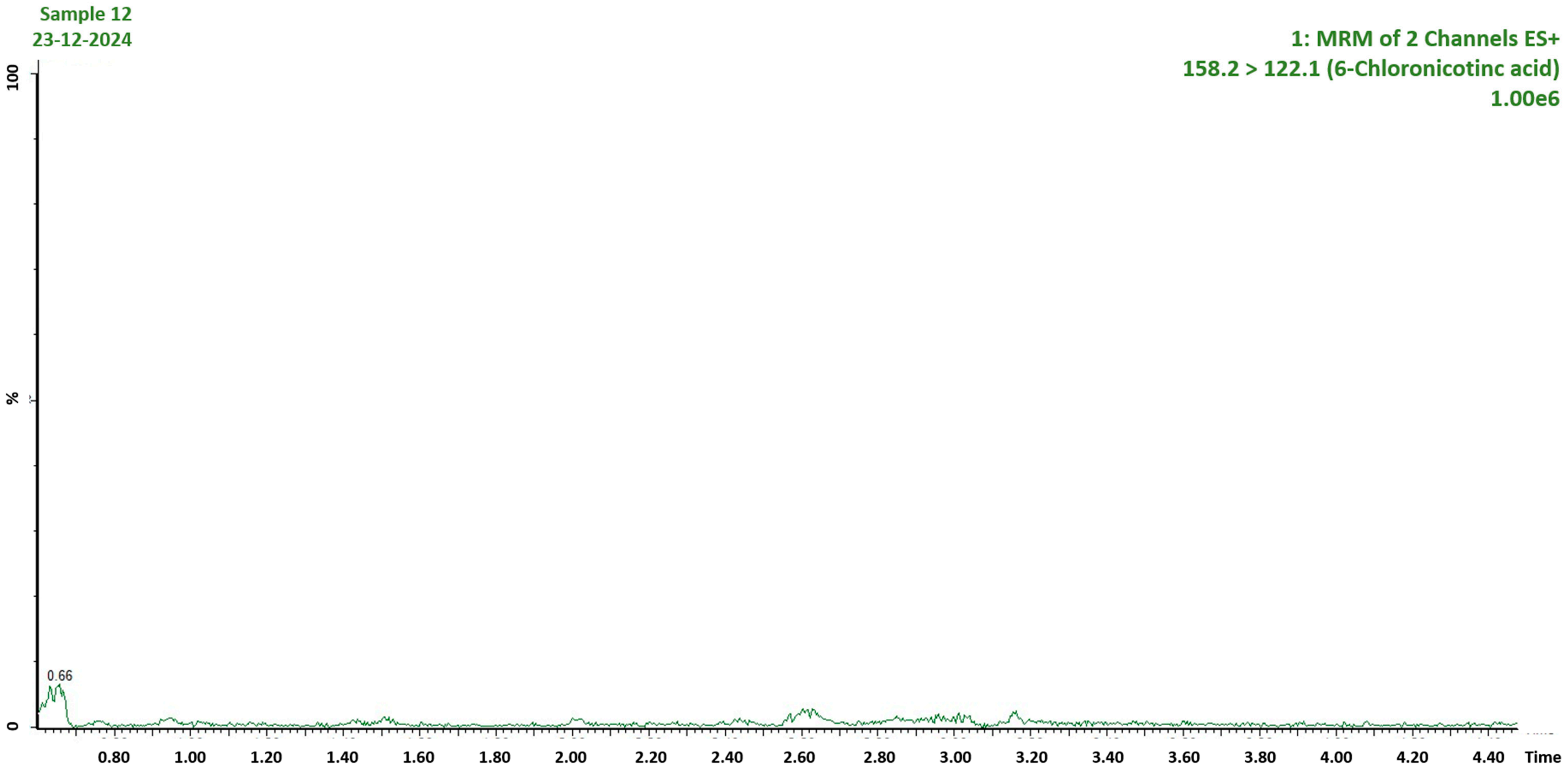

- Imidacloprid-olefin

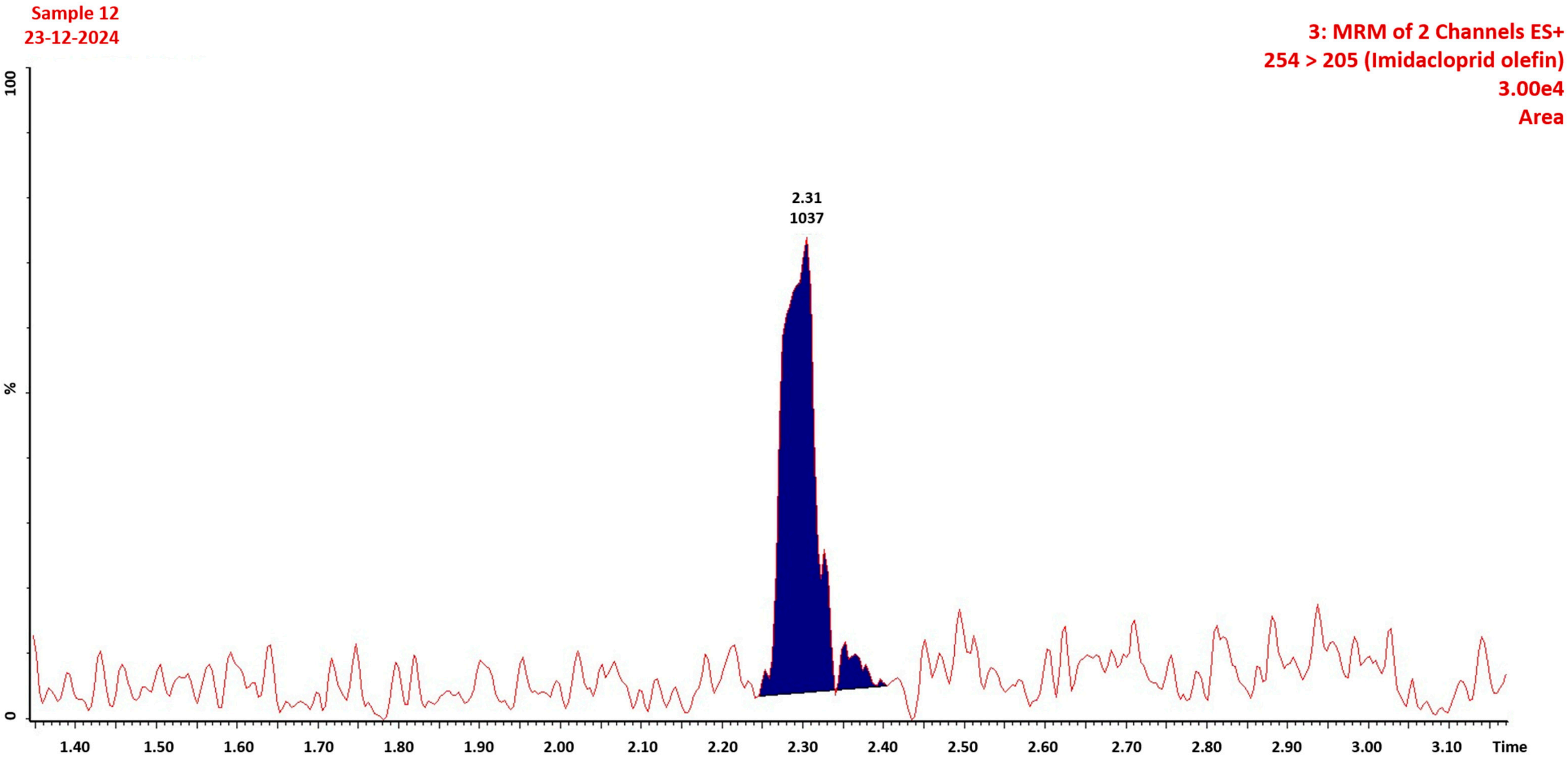

- **5-Hydroxy-imidacloprid**

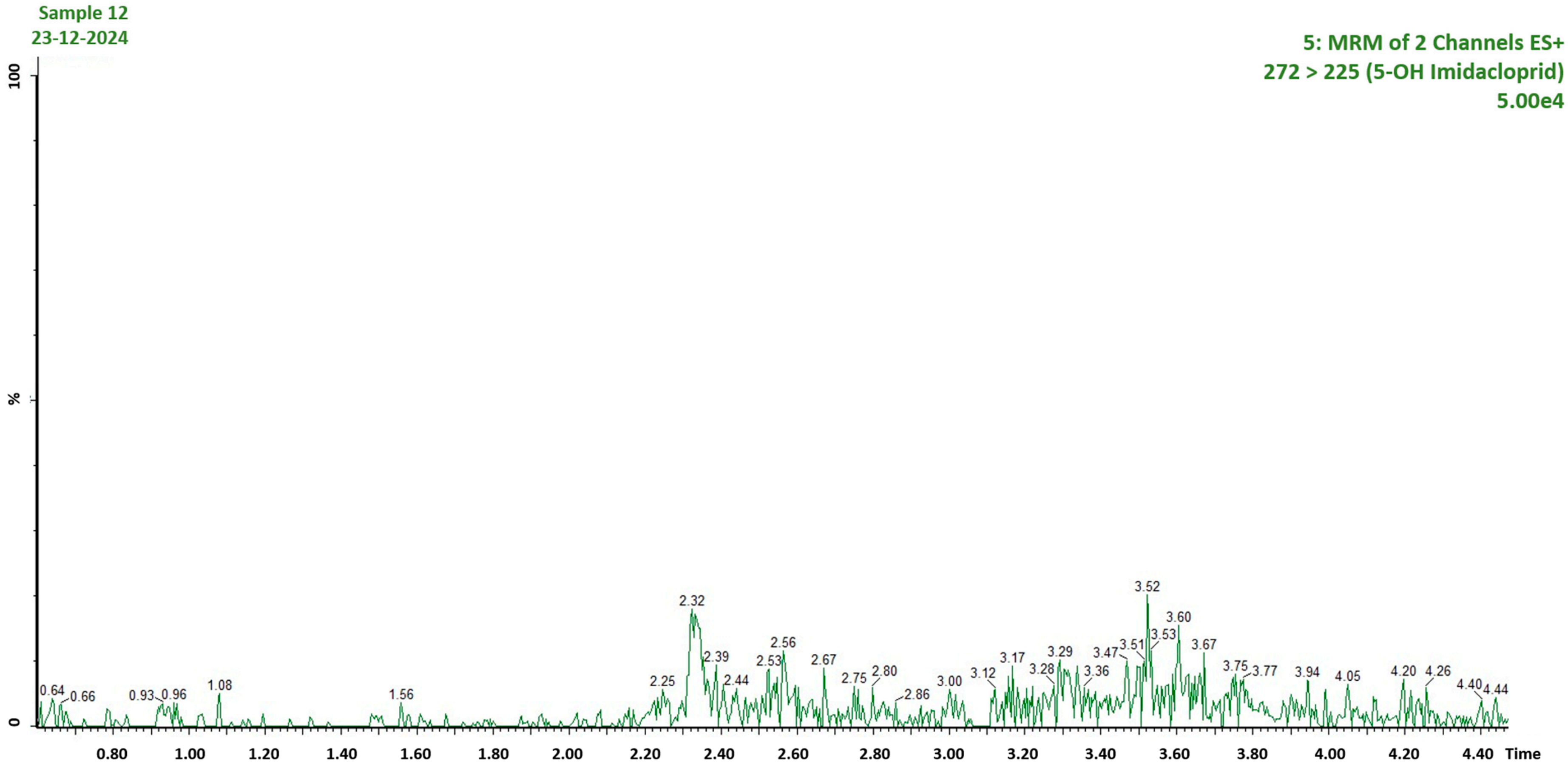

4. Imidacloprid (Commercial quality) after fungal treatment

- 6-chloronicotinic acid

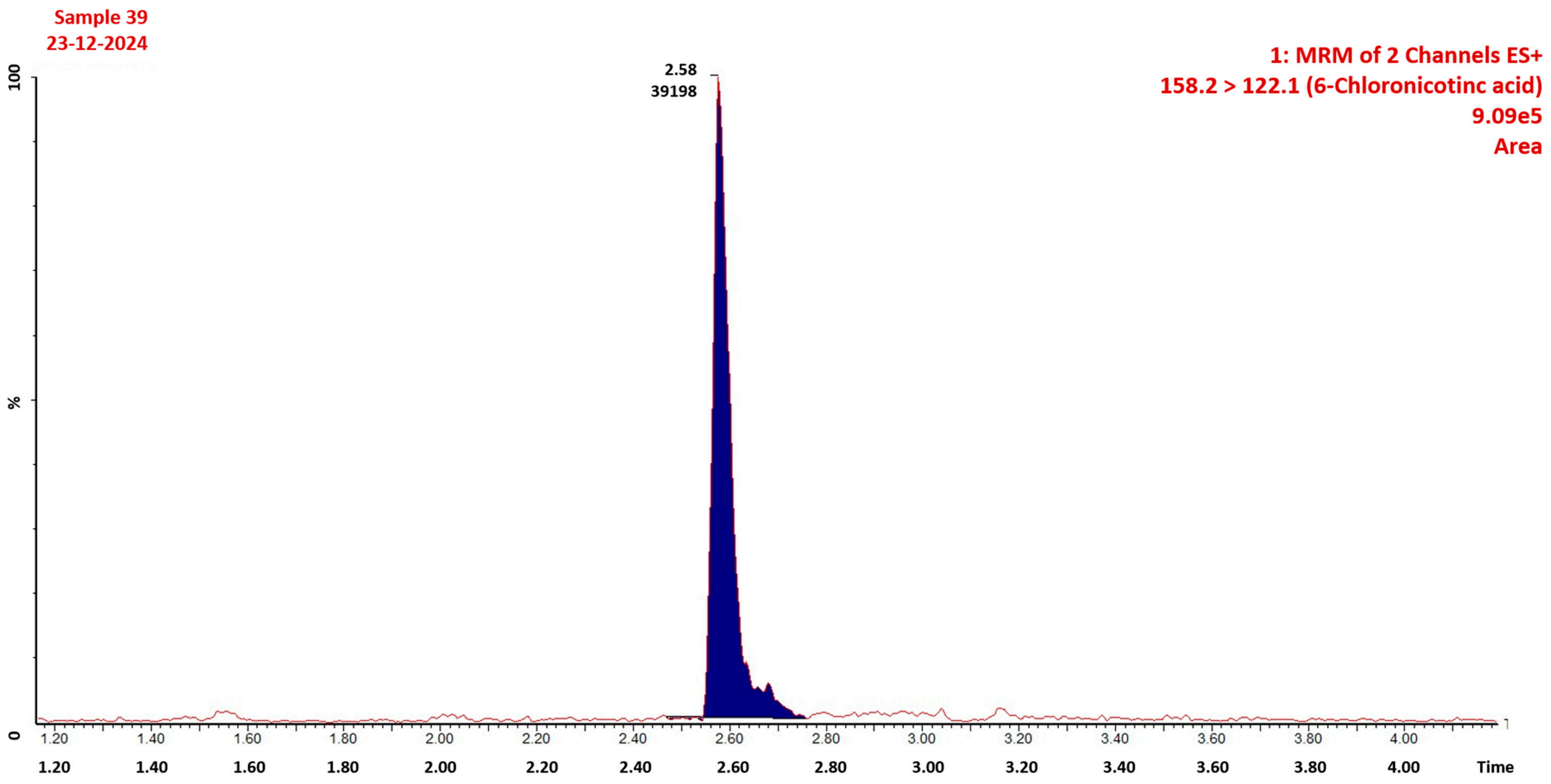

- **Imidacloprid-olefin**

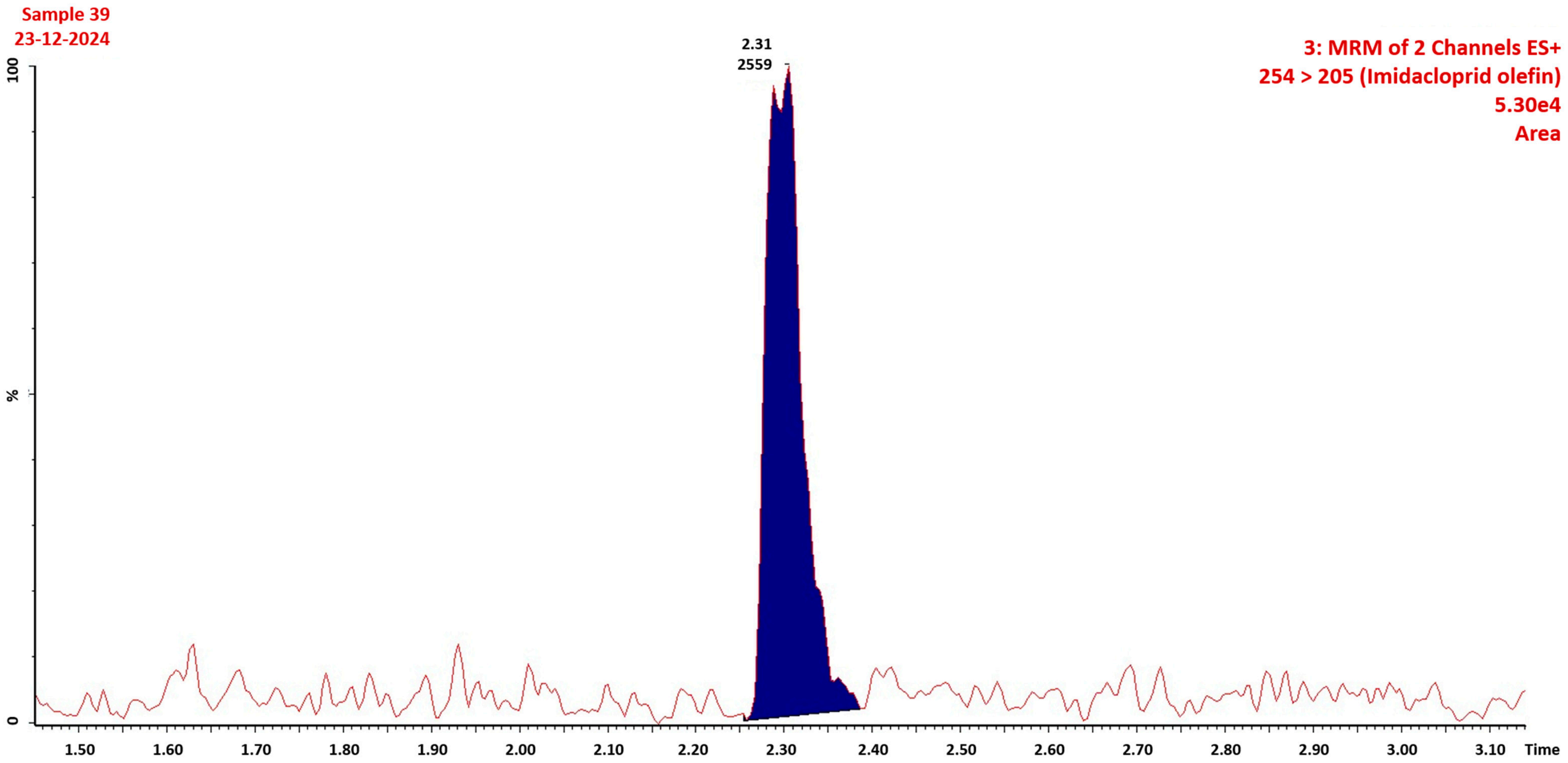

- **5-hydroxy- imidacloprid**

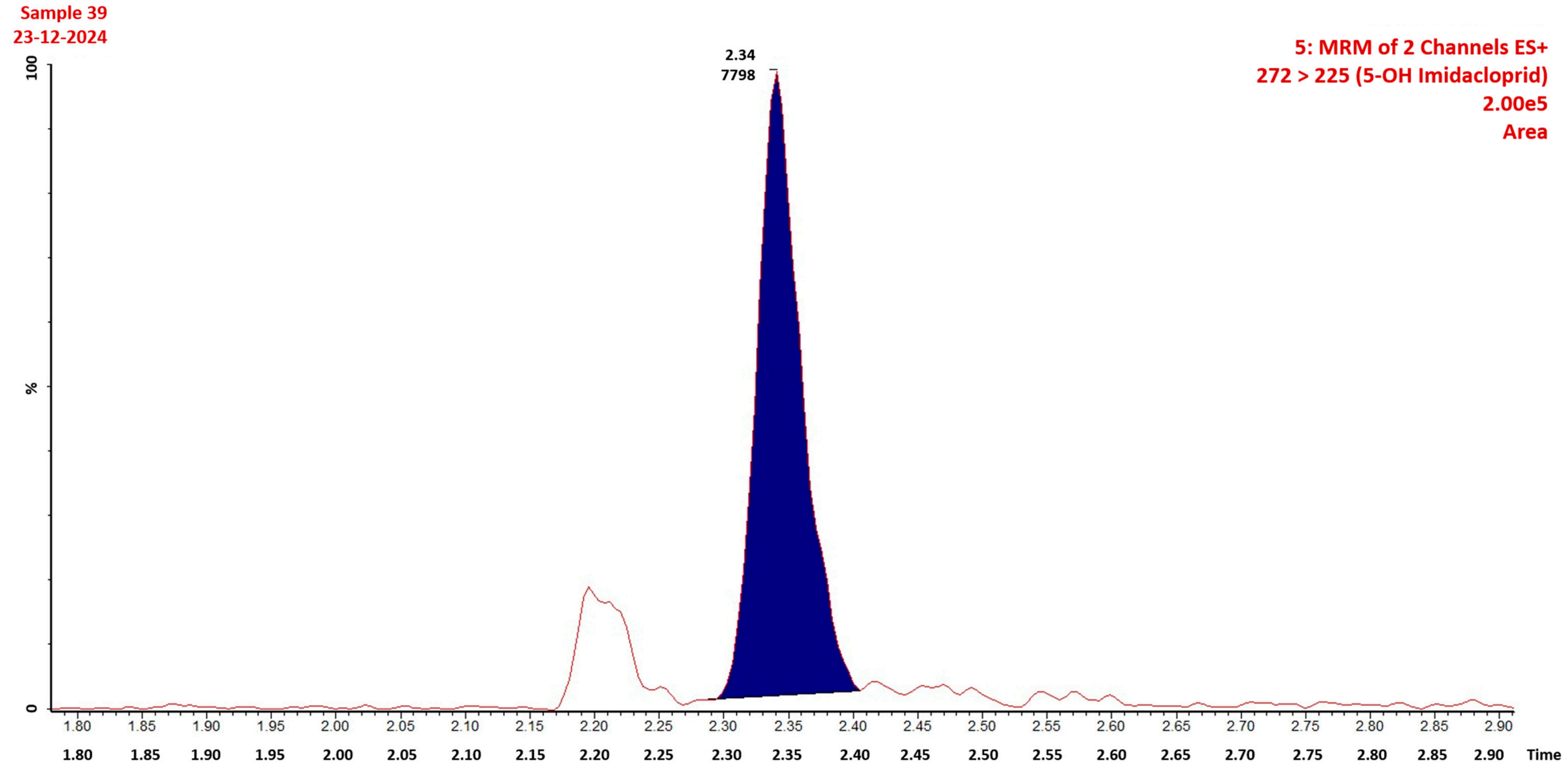

Supplement: Supplementary file 1 [file jof-12-00724-s001.zip › jof-4540590-supplementary.pdf]
